# Brain metastasis‐associated fibroblasts shape the tumour microenvironment to enhance cancer cell invasion

**DOI:** 10.1111/bpa.70129

**Published:** 2026-08-04

**Authors:** Barbora Výmolová, Lucie Pfeiferová, Tadeáš Karel Smetana, Petr Výmola, Nikola Ternerová, Michal Zubaľ, Michal Kolář, Jana Šáchová, Soňa Gyönyörová, Eva Balážiová, Magdalena Houdová Megová, Jakub Červenka, Helena Kupcová Skalníková, Michal Španko, Karel Smetana, Lukáš Lacina, David Netuka, Robert Tomáš, Petr Bušek, Aleksi Šedo

**Affiliations:** ^1^ Laboratory of Cancer Cell Biology, Institute of Biochemistry and Experimental Oncology, First Faculty of Medicine Charles University Prague 2 Czech Republic; ^2^ Laboratory of Genomics and Bioinformatics Institute of Molecular Genetics of the Czech Academy of Sciences Prague 4 Czech Republic; ^3^ Laboratory of Proteomics, Institute of Biochemistry and Experimental Oncology, First Faculty of Medicine Charles University Prague 2 Czech Republic; ^4^ Institute of Anatomy, First Faculty of Medicine Charles University Prague 2 Czech Republic; ^5^ Department of Neurosurgery and Neurooncology, First Faculty of Medicine Charles University and Military University Hospital Prague 6 Czech Republic; ^6^ Department of Neurosurgery Motol and Homolka University Hospital Prague 5 Czech Republic; ^7^ Present address: Protein Chemistry, Department of Translational Medicine, Faculty of Medicine Lund University Malmö Sweden; ^8^ Present address: Department of Oncology, Second Faculty of Medicine Charles University and Motol and Homolka University Hospital Prague 5 Czech Republic

**Keywords:** brain metastases, cancer‐associated fibroblasts, cell migration, extracellular matrix, primary cell culture, transcriptome

## Abstract

Brain metastases (BrM) are a frequent and life‐threatening complication of solid tumours, with lung cancer representing their most common source. While cancer‐associated fibroblasts (CAFs) are well‐established contributors to tumour progression in many extracranial malignancies, their presence and function within the brain tumour microenvironment, where fibroblasts have long been considered scarce or absent, remain poorly understood. Here, we isolated and characterised fibroblast‐like cells from 13 human BrMs of diverse origins. These BrM‐associated CAFs (BrM‐CAFs) expressed canonical CAF markers and showed reduced proliferation and increased senescence compared to normal fibroblasts. Their transcriptome was enriched for extracellular matrix (ECM)‐related genes, including multiple collagens, fibronectin, and matrix‐remodelling enzymes. In vitro, BrM‐CAFs produced a fibrillar ECM, and in BrM tissues, their abundance was associated with collagen I and fibronectin deposition. Transcriptomic, proteomic, and secretome analyses further revealed that BrM‐CAFs produce multiple cytokines, chemokines, and growth factors that promote cell motility. BrM‐CAF conditioned medium promoted both monocyte migration and the migration of cancer cells, including established cell lines and patient‐derived lung cancer BrM cells; monocyte migration was partially reduced by inhibition of CCL2/CCR2 signalling, whereas blocking CXCL12, CXCL16, or CX3CL1 attenuated BrM‐CAF‐induced cancer cell migration. Beyond these effects on migration, exposure to BrM‐CAFs increased cancer cell invasion in transwell and heterotypic 3D spheroid assays. In contrast, their effects on cancer cell proliferation were limited and did not indicate a growth‐promoting role. Exposure to BrM‐CAFs was also associated with increased expression of interferon‐stimulated genes in cancer cells. Together, our findings support a role for BrM‐CAFs in shaping the brain metastatic microenvironment through ECM remodelling and the secretion of pro‐migratory mediators, promoting monocyte and cancer cell migration and enhancing cancer cell invasion. These data identify BrM‐CAFs as active stromal participants in BrM biology and support further investigation of their biological, diagnostic, and therapeutic relevance.

## Graphical abstract

Brain metastasis‐associated fibroblasts are active stromal components of the metastatic niche characterised by extracellular matrix remodelling and production of pro‐migratory mediators. They promote monocyte and cancer cell migration and enhance cancer cell invasion while having no growth‐promoting effect on cancer cells.

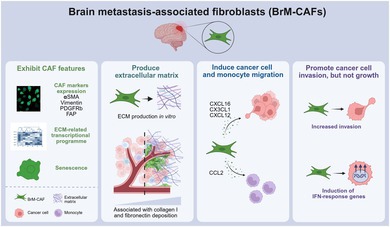

## 1 INTRODUCTION

Brain metastases (BrM), which occur in up to 40% of patients with metastatic cancers, represent the most common brain malignancy in adults and are associated with a poor prognosis because of the limited effectiveness of available therapeutic options [1]. Given their high clinical relevance, BrMs have become a focus of intensive research, particularly to elucidate the molecular mechanisms underlying their establishment and progression, with the ultimate goal of developing new therapeutic approaches.

The brain constitutes a unique microenvironment, distinct in its cellular and noncellular components as well as its metabolic and immunological landscapes, which poses a challenge for metastasising cancer cells to settle, adapt, and prosper [2]. Because of these specific features, the insights from primary extracranial tumours cannot be directly extrapolated to BrMs. According to the “seed and soil” hypothesis, metastatic progression depends not only on the intrinsic properties of cancer cells (the “seed”) but also on a supportive microenvironment (“soil”), which includes various types of stromal cells [3, 4].

Cancer‐associated fibroblasts (CAFs) are a well‐established component of the tumour microenvironment in extracranial malignancies, where they promote tumour progression through various mechanisms, including stimulation of cancer cell growth, angiogenesis, extracellular matrix (ECM) remodelling, and immune modulation [5, 6]. Mechanistically, CAFs act via the secretion of ECM components and multiple soluble mediators [7]. Under prolonged tumour microenvironment stress, CAFs may become senescent [8], adopting a protumorigenic senescence‐associated secretory profile (SASP) [9] characterised by the production of numerous secreted molecules that contribute to an immunosuppressive landscape and promote migration and invasion of cancer cells [10, 11, 12]. Because of their heterogeneity and the absence of a specific marker, CAFs are typically identified using a combination of markers, such as vimentin, PDGFRα/β, αSMA, and fibroblast activation protein (FAP) [13]. Among these, FAP stands out because of its selective expression in tumour‐associated stroma in diverse malignancies and its emerging diagnostic and therapeutic potential, including in brain tumours [14, 15].

CAFs have been extensively characterised in extracranial malignancies and have been implicated in metastatic dissemination and outgrowth [16, 17]. While their presence in the brain was once questioned, recent studies—including our own—have identified CAFs in the BrM microenvironment [18, 19, 20]. Because of their frequent FAP positivity, BrM‐associated CAFs (BrM‐CAFs) can serve as diagnostic or therapeutic targets in BrMs of various origins [19]. In addition, they likely contribute to shaping the metastatic niche. Transcriptomic studies of breast and lung cancer BrMs have highlighted an active crosstalk between fibroblast‐like cells, cancer cells, and other stromal populations in the BrM microenvironment [21, 22]. However, their precise role remains poorly understood.

We hypothesise that BrM‐CAFs actively shape the BrM microenvironment through multiple mechanisms, such as ECM remodelling and secretion of bioactive soluble mediators. To test this, we derived BrM‐CAFs from BrMs of various origins, characterised their phenotype, transcriptome, and secretome, and investigated their interactions with cancer cells. Focusing particularly on lung cancer BrMs—the most prevalent type—our objective was to decipher the role of BrM‐CAFs and their contribution to the brain metastatic niche.

## 2 MATERIALS AND METHODS

### 2.1 Patient characteristics

Tumour tissue samples and non‐tumorous brain or subgalear connective tissue samples were collected during neurosurgical resection of BrMs (*n* = 64), glioblastoma (GBM; *n* = 6), pharmacoresistant epilepsy (PRE, mesiotemporal sclerosis; *n* = 6), or meningioma (*n* = 1) at the Department of Neurosurgery, Homolka, Motol and Homolka University Hospital, Prague and the Department of Neurosurgery and Neurooncology, First Faculty of Medicine, Charles University and Military University Hospital, Prague. Sample collection was approved by the respective institutional Ethics Committees and written informed consent was obtained from all donors prior to surgery. Patient characteristics, the origin of the BrMs (when available), and the analyses performed on each sample are summarised in Table S1, Supporting Information. For tissue lysate preparation and immunohistochemical analyses, snap frozen tissue samples stored at −80°C were used. For primary cell culture derivation, tissue samples were collected in Dulbecco's modified Eagle's medium/Nutrient Mixture F‐12 (DMEM F12) supplemented with 100 units/mL penicillin G and 100 μg/mL streptomycin (P/S) and processed as described below.

### 2.2 Derivation of primary fibroblast cultures and BrM‐derived cancer cells

Fresh tissue samples from BrM, GBM, PRE, or subgalear connective tissue were cleared of macroscopic vessels and necrosis, minced to ~1 mm^3^ pieces, and subjected to combined mechanical and enzymatic dissociation using the Tumour Dissociation Kit and gentleMACS Dissociator (Miltenyi) or the Papain Dissociation Kit (Worthington; used only for GBM‐derived CAFs) according to the manufacturer's instructions. The suspension was then cleared of debris using Debris Removal Solution, and erythrocytes were lysed with Red Blood Cell Lysis Solution (10×) (both Miltenyi). The resulting cell suspension was cultured in DMEM supplemented with 10% fetal calf serum (FCS) and 1% P/S to expand the cells. For GBM‐derived CAFs, the medium was further supplemented with 1% GlutaMAX (Life Technologies), 1% sodium pyruvate (Sigma‐Aldrich), and 1% non‐essential amino acid (NEAA) solution (Sigma‐Aldrich). Magnetic‐activated cell sorting (MACS) using Fibroblast Microbeads (Miltenyi) was used to isolate fibroblasts in all cases. For the isolation of the BrM‐derived cancer cell culture NCH724, similar tissue processing was performed, followed by expansion of the non‐fibroblast population (i.e., the negative fraction of MACS). The phenotype of the isolated populations was verified by immunocytochemistry as described below. Normal dermal fibroblasts were isolated and characterised at the Institute of Anatomy, Charles University in Prague, from facial skin biopsies of young adults following a previously described protocol [23].

### 2.3 Cell culture, preparation of conditioned media and cell lysates

Cell lines A549 (European Collection of Cell Cultures) and LXF289 (German Collection of Microorganisms and Cell Cultures) were cultured in DMEM +10% FCS. All patient‐derived cell cultures (BrM‐derived cancer cells, BrM‐CAFs, GBM‐CAFs, PRE‐derived normal fibroblasts [NF], Subgalear‐NF) were cultured in DMEM +10% FCS + P/S and used up to passage 5 after isolation. All cells were cultured in a humidified atmosphere at 37°C and 5% CO_2_, and the absence of mycoplasma contamination was routinely verified. To prepare BrM‐CAF conditioned media (CM), 4 × 10^5^ cells were seeded into a 100‐mm Petri dish, grown until subconfluent, and incubated in serum‐free medium for 48 h. The CM was collected, clarified by centrifugation (10 min, 600*g*, 4°C) and filtration (0.22 μm pore filters), and stored at −80°C until further use. For transcriptomic profiling, 4 × 10^5^ cells were seeded into a 100‐mm Petri dish, grown until subconfluent, harvested in 350 μL of RLT buffer (Qiagen), and stored at −80°C until RNA was isolated as described below.

For proteomic analyses, cell lysates were prepared from six cultures (BrM‐CAF *n* = 3 and Subgalear‐NF *n* = 3). Cells (4 × 10^5^) were seeded into a 100‐mm Petri dish and grown until subconfluent. Then, the culture medium was aspirated, and the cell monolayer was washed twice with pre‐warmed (37°C) phosphate‐buffered saline (PBS). Cells were then lysed by adding a lysis buffer (1% sodium dodecyl sulphate in 50 mM Tris–HCl, pH 7.5), pre‐heated to 95°C. Complete cell lysis was immediately verified under a microscope. Subsequently, the lysates were scraped, collected into microtubes, and incubated for an additional 5 min at 95°C to ensure complete protein denaturation. Finally, the samples were stored at −80°C until further use.

### 2.4 Preparation of fluorescently labelled cancer cells

To prepare fluorescently labelled A549 (A549LucmKate) or NCH724 (NCH724LucmKate), we followed a previously described protocol [24]. We used pseudolentiviral particles produced in HEK293FT cells (Thermo Fisher Scientific) using jetPRIME DNA and siRNA transfection reagent (VWR). Briefly, for each 75 cm^2^ flask of HEK293FT cells, a mixture of jetPRIME buffer (1.5 mL), DNA vectors of interest (pRSV‐Rev, pMDLg/pRRE, pMD2.G (Addgene) and pLV[Exp]‐Puro‐CAG>Luc2(ns):P2A:mKate2 (Vectorbuilder); 1.5 pmol of DNA each), and jetPRIME reagent (90 μL) was prepared, centrifuged (2 min, 300*g*), and incubated (30 min at room temperature, RT). This mixture was added to 10 mL of fresh culture medium and incubated with HEK293FT cells for 16 h. Afterwards, the medium was removed, 20 mL of fresh culture medium was added, and viral production was allowed to proceed for 72 h. Finally, the pseudolentiviral particle‐containing medium was collected, centrifuged (5 min, 300*g*), and filtered (0.45 μm PES filter), and Polybrene (8 μg/mL, Merck) was added. The medium containing lentiviral particles was incubated with A549 or NCH724 cells overnight. After replacement with fresh culture medium and an additional 48 h, puromycin (2 μg/mL) was used for the selection of transduced cells. Flow cytometry confirmed mKate fluorescence in 100% of the selected cell population.

### 2.5 Senescence assay

Senescent cells were detected using the Senescence Cells Histochemical Staining Kit (Sigma‐Aldrich), based on the detection of β‐galactosidase activity, according to the manufacturer's instructions. Briefly, 13 × 10^3^ BrM‐CAF or Subgalear‐NF cells per well were seeded in 24‐well plates (WP). After 4 days of growth, cells were washed twice with PBS and fixed using the Fixation Solution. After three further washes with PBS, the Staining Mixture was applied. The staining reaction was allowed to proceed overnight, resulting in a blue signal indicative of β‐galactosidase activity. Total cell count and β‐galactosidase+ cell count were determined in five visual fields per well, and each cell culture was analysed in quadruplicate. Total cell count served as a measure of cell growth; the percentage of β‐galactosidase+ cells was used to estimate the level of cellular senescence.

### 2.6 Production of 3D extracellular matrices

The deposition of ECM in vitro was evaluated by generating matrices as described previously [25]. First, a coverslip was coated with 0.2% cold water fish gelatine (Sigma‐Aldrich; coating for 1 h at 37°C). After a wash with PBS, 1% glutaraldehyde was added (30 min at RT) for crosslinking the gelatine. The glutaraldehyde was removed by three PBS washes, one wash with 1M ethanolamine (30 min at RT) and three more PBS washes. Finally, 2 mL of DMEM +10% FCS + P/S + 50 μg/mL ascorbic acid were added, and 5 × 10^5^ BrM‐CAF cells were seeded. To produce the extracellular matrices, the cells were grown for 8 days in total, with medium changes every 2 days, and eventually decellularised using an alkaline detergent treatment (0.5% Triton X‐100, 20 mM NH_4_OH in PBS for 5 min at 37°C).

### 2.7 Cancer cell migration assay

The transwell migration assay was performed as follows. 45 × 10^3^ cancer cells (A549, LXF289, NCH724) in 100 μL of serum‐free DMEM were placed in the upper chamber of transwell inserts for 24WP (8.0 μm pores, Corning). Six‐hundred microlitre of the respective medium (CM from BrM‐CAF or Subgalear‐NF, control medium) was placed in the lower chamber as a chemoattractant. For experiments using CXCL16‐ or CX3CL1‐blocking antibodies, the respective blocking antibody or a non‐specific immunoglobulin control was added to the BrM‐CAF CM, each at a concentration of 900 ng/mL. For experiments using the CXCR4 inhibitor, the CXCR4 inhibitor (AMD3100/Plerixafor, 2.5 μM) was pre‐incubated with cancer cell suspension for 2 h and also added to the BrM‐CAF CM used as chemoattractant. The cells were allowed to migrate for 18 h. Then, the non‐migrating cells in the upper chamber were removed with a cotton swab. Migrating cells were fixed with 4% paraformaldehyde and stained with crystal violet (Sigma‐Aldrich). The number of migrating cells was evaluated using Fiji (ImageJ, NIH) in five visual fields (200× magnification) per insert. All experiments were performed three times in triplicate.

### 2.8 Cancer cell and BrM‐CAF indirect coculture and transwell invasion assay

To evaluate the impact of BrM‐CAFs on cancer cell invasion, indirect cocultures were established using transwell inserts for 6WP (0.4 μm pore size, Corning). BrM‐CAFs (117 × 10^3^) were seeded onto the lower side of the inserts and allowed to adhere for 24 h. Inserts were then placed upright in 6‐well plates with complete medium, and A549 cells (117 × 10^3^) were seeded on the upper (apical) side. The cocultures were maintained for 48 h without medium exchange. A549 monocultures were processed in parallel as controls. After coculture, A549 cells were collected, resuspended in serum‐free DMEM (40 × 10^3^ cells/mL), and seeded into Matrigel‐coated invasion inserts (8 μm pore size, Corning) in 24‐well plates. The lower chamber contained DMEM +10% FCS as a chemoattractant. The cells were allowed to invade for 24 h. Non‐invading cells were then removed from the upper side using a cotton swab. The inserts were fixed with 4% paraformaldehyde for 10 min, stained with crystal violet (Sigma‐Aldrich), and air‐dried. Invading cells were imaged using a 10× objective and manually quantified in five visual fields per insert using Fiji (ImageJ, NIH).

### 2.9 3D invasion assay

The 3D invasion assay in the collagen matrix was performed according to a previously described protocol [26] with minor modifications. Briefly, spheroids composed of 1.5 × 10^3^ fluorescent cancer cells (A549LucmKate) alone or in combination with 1.5 × 10^3^ unlabelled BrM‐CAFs were prepared using agarose moulds formed in MicroTissue 3D petri dish (Merck) according to the manufacturer's protocol and allowed to form for 72 h. A 1.5 mg/mL collagen I (Gibco) solution in DMEM +10% FCS, neutralised with 1M NaOH, was used to form a collagen base layer in 24WP (200 μL per well). After the collagen had polymerised (approximately 30 min), the spheroids were harvested, resuspended in the same collagen I solution, and seeded onto the collagen base layer (500 μL containing approximately 20 spheroids per well in 24WP). After an additional 60 min, 250 μL of DMEM +10% FCS was added per well to the collagen I matrix. The spheroids were allowed to invade the collagen matrix for 96 h; images were acquired every 24 h using the BioTek Cytation 5 Reader and Imager (Agilent) in TexasRed and bright‐field channels. The area covered by fluorescent cells was determined using Gen5 software. For each spheroid, the core area measured 24 h after was used for normalisation of all subsequent measurements.

### 2.10 Longitudinal monitoring of cancer cell growth in coculture

To evaluate cancer cell growth in coculture with BrM‐CAFs, 1 × 10^3^ A549LucmKate or NCH724LucmKate cells, with or without the addition of BrM‐CAFs (cancer cell:CAF ratios of 1:4, 1:1, and 4:1), were seeded per well in 96WP. Cells were allowed to adhere for 24 h, after which the number of fluorescent cancer cells was monitored over the subsequent 72 h using a BioTek Cytation 5 Reader and Imager (Agilent). Images were acquired every 8 h from one visual field per well using a 4× objective, enabling selective quantification of cancer cells independently of cocultured CAFs. Cell counts were determined using Gen5 software (Agilent).

### 2.11 EdU incorporation assay

A549LucmKate proliferative activity was assessed using a 5‐ethynyl‐2′‐deoxyuridine (EdU) incorporation assay followed by flow cytometric analysis. A549LucmKate cells were cultured either alone or in coculture with patient‐derived BrM‐CAFs (NCH402 or NCH621) in 60 mm Petri dishes for 48 h in DMEM supplemented with 10% FCS. For coculture experiments, cancer cells and fibroblasts were seeded at a ratio of 1:4 (65 × 10^3^ cancer cells and 260 × 10^3^ fibroblasts per dish). EdU (Carl Roth) was added to the culture medium at a final concentration of 10 μM during the final 60 min of culture. Cells were harvested by trypsinisation, washed with PBS, and fixed with 4% paraformaldehyde for 10 min at RT. Cells were subsequently permeabilised with 0.1% Triton X‐100 (SERVA Electrophoresis GmbH) for 10 min and washed with PBS containing 1% bovine serum albumin (BSA, Sigma‐Aldrich). EdU incorporation was detected by click chemistry using 6‐carboxyfluorescein azide (Carl Roth; final concentration 10 μM), CuSO_4_·5H_2_O (Penta Chemicals Unlimited; final concentration 2 mM), and sodium ascorbate (Carl Roth; final concentration 10 mM). Cells were incubated with the reaction mixture for 30 min at RT in the dark and subsequently washed with PBS containing 1% BSA. Flow cytometric analysis was performed using a NovoCyte flow cytometer (Agilent Technologies). Cancer cells were identified based on mKate fluorescence following exclusion of debris and doublets. EdU incorporation was quantified as the percentage of EdU‐positive cells within the mKate‐positive cancer cell population.

### 2.12 Cancer cell and BrM‐CAF direct coculture and fluorescence‐activated cell sorting (FACS)

To assess transcriptomic changes in cancer cells induced by direct cell‐to‐cell contact with BrM‐CAFs, 100 mm Petri dishes were seeded with 3 × 10^5^ fluorescently labelled cancer cells (A549LucmKate) together with 3 × 10^5^ BrM‐CAFs (NCH621 or H255). The CAFs were labelled with Tag‐it Violet™ Proliferation and Cell Tracking Dye (Biolegend) according to the manufacturer's instructions. Cancer cells and BrM‐CAFs were cocultivated for 72 h. The cells were then harvested, centrifuged, resuspended in sorting buffer (5% FCS + 1% ethylenediaminetetraacetic acid [EDTA] in PBS) at a concentration of 2.5 × 10^6^ cells/mL, and filtered through a cell strainer with 40 μm pores (VWR). Cancer cells were isolated using the MA900 Multi‐Application Cell Sorter (SONY), followed by centrifugation (300*g*, 5 min, 4°C) and resuspension in 400 μL of PBS. The purity of the sorted populations was verified using a Countess 3 Automated Cell Counter (Thermo Fisher Scientific) and exceeded 98% in all cases. After additional centrifugation (300*g*, 5 min, 4°C), cells were lysed in 350 μL/sample of Buffer RLT (Qiagen) and stored at −80°C for subsequent RNA isolation as described below.

### 2.13 RNA‐sequencing and transcriptome analysis

Total RNA was isolated from cell cultures using RNeasy Micro Kit (Qiagen) according to the manufacturer's protocol, including treatment by DNase I. The quantity and quality of the isolated RNA were measured by a NanoDrop ND‐1000 (Thermo Fisher Scientific) and an Agilent 2100 Bioanalyzer (Agilent Technologies, RNA 6000 Nano chip). Only samples with RNA integrity number greater than 7 were used for further analysis. For BrM‐CAF characterisation, RNA sequencing libraries were prepared from 1 μg of total RNA using the KAPA mRNA HyperPrep Kit, including polyA selection and barcoding with the KAPA UDI Adapter Kit (all Roche). For cancer cells cocultured with BrM‐CAFs, libraries were prepared from 0.5 μg of total RNA using the NEBNext Ultra II Directional RNA Lib Prep kit for Illumina (New England BioLabs). For both experiments, library size distribution and concentration were determined using the Agilent 2100 Bioanalyzer (Agilent Technologies, High Sensitivity DNA chip) and a Qubit 2.0 Fluorometer (Thermo Fisher Scientific), respectively. Libraries were pooled at equimolar ratios, and those from cocultured cancer cells were additionally cleaned with SPRIselect beads (Beckman Coulter). Sequencing was performed on a NextSeq 500 platform (Illumina) using a 75‐bp single‐end configuration.

The quantification of detected genes and the technical quality control were performed with the nf‐core/rnaseq v3.4 bioinformatics pipeline [27] with STAR mapping [28] and Salmon quantification [29]. GRCh38 (ensEMBL assembly version 104) served as the reference genome [30]. Genes detected in a single sample were excluded from further analysis.

Differential gene expression analysis was performed using the DESeq2 R package (v1.38.3) [31] within the Bioconductor framework (v3.16). Genes were considered significantly differentially expressed if they exhibited a minimum twofold change in expression levels and a false discovery rate (FDR) below 0.1. To improve log‐fold change (LFC) estimates, the adaptive shrinkage method described in [32] was applied. Reference expression profiles of astrocytes, endothelial cells, microglia, and T‐lymphocytes were obtained from [33]. Only data from control/healthy samples were used and processed standardly in the Seurat v 5.1 R package using default parameters [34]. Heatmaps were created using the ComplexHeatmap package [35] based on standardised log2‐transformed, quantile‐normalised transcripts per million (TPM) values, and corresponding normalised values from Seurat for reference expression profiles (z‐score).

Gene Ontology (GO) overrepresentation analysis was performed separately for upregulated and downregulated DEGs using Enrichr (https://maayanlab.cloud/Enrichr/enrich). To reduce redundancy among significantly enriched GO terms, results were summarised using Revigo (http://revigo.irb.hr/ [36]) based on semantic similarity. GO terms with a dispensability score <0.5 for Biological Processes and <0.1 for Cellular Components were retained for further interpretation and visualisation.

Candidate migration‐regulating mediators were identified among upregulated DEGs by selecting genes associated with the GO Biological Process terms “Regulation of cell migration” and “Positive regulation of cell migration.”

The transcriptomic datasets used in this article are available in the ArrayExpress database (https://www.ebi.ac.uk/biostudies/arrayexpress) under the accession numbers E‐MTAB‐13242 and E‐MTAB‐15451.

### 2.14 Liquid chromatography‐mass spectrometry sample preparation, analysis, and data processing

Cell lysates from BrM‐CAFs (H131, NCH402, and NCH621) and Subgalear‐NFs (NCH233, NCH379, and NCH434) were processed using a modified filter‐aided sample preparation (FASP) protocol [25] with minor modifications. Briefly, Microcon 30K centrifugal ultrafiltration units (Merck) were pre‐washed with water, and 30 μg of protein per sample were loaded onto the units. The ultrafiltration units were then washed three times with a wash buffer (8M urea and 5 mM EDTA in 50 mM ammonium bicarbonate) by centrifugation at 13,000*g* for 20 min at 22°C. Subsequently, proteins were reduced with 10 mM tris(2‐carboxyethyl) phosphine in the wash buffer (30 min at 32°C) and alkylated with 40 mM iodoacetamide in the wash buffer (30 min at 25°C, in the dark). Finally, the ultrafiltration units were washed once with the wash buffer and three times with 50 mM ammonium bicarbonate, and centrifuged (14,000*g*, 25 min, 22°C). On‐filter digestion was performed with 0.6 μg of rLys‐C protease (Promega) and 1 μg of Trypsin Platinum protease (Promega) in 50 mM ammonium bicarbonate containing 0.02% (w/v) ProteaseMAX (Promega) overnight at 37°C. Peptides were collected by centrifugation, acidified by formic acid to a final concentration of 2% (v/v), and centrifuged (14,000*g*, 20 min, 4°C). The supernatants were desalted using Pierce peptide desalting spin columns (Thermo Scientific) according to manufacturer's instructions, except formic acid was used instead of trifluoroacetic acid. Peptides were dried using a vacuum centrifuge at 45°C and resuspended in 0.1% formic acid in water. Peptide concentrations were measured at *λ* = 280 nm (Synergy HTX, BioTek). Samples were then diluted to 0.2 μg/μL in QuanRecovery with MaxPeak HPS vials (Waters) and spiked with indexed Retention Time (iRT) peptides (Biognosys) at iRT:sample ratio 1:30 (v/v).

Peptide samples were loaded onto a trap column (PepMap Neo C18, 5 mm × 0.3 mm, 5 μm, Thermo Scientific) and subsequently separated on an analytical column (PepSep Ultra C18, 25 cm × 75 μm, 1.5 μm, Bruker Daltonics) using a linear gradient of 2–35% acetonitrile with 0.1% formic acid over 90 min at a flow rate of 200 nL/min. The analytical column was maintained at 50°C. Liquid chromatography was performed using a nanoElute 2 system (Bruker Daltonics) coupled to a mass spectrometer timsTOF HT (Bruker Daltonics) with a CaptiveSpray 2 ion source. For data‐dependent acquisition (DDA), 0.6 μg of peptides per sample was analysed with the default method DDA PASEF‐standard_1.1_sec_cycletime (1/K0 range of 0.6–1.6 V·s/cm^2^, ramp/accumulation time of 100 ms, 10 PASEF ramps per cycle, and a total cycle time of 1.17 s) employed in positive ion mode with adjusted MS scan range of 300–1700 m/z. For data‐independent acquisition (DIA), 0.6 μg of peptides per sample was analysed with the default method dia‐PASEF‐long gradient (1/K0 range of 0.6–1.6 V·s/cm^2^, ramp/accumulation time of 100 ms, 16 MS/MS ramps, 32 MS/MS windows, and a cycle time of 1.8 s) employed in positive ion mode with adjusted MS scan range of 300–1700 m/z.

Raw data were analysed in Spectronaut (version 19.5.241126.62635, Biognosys). DIA data were processed by directDIA analysis workflow with default BGS Factory Settings with the following modifications. The corresponding DDA data were employed as library extension runs to create a hybrid spectral library. The protein FASTA database was compiled by merging the UniProtKB Homo sapiens reference proteome (release 2024_06, proteome updated on 07102024; one protein sequence per gene, in total 20,650 proteins) [37], with the 381 universal contaminant proteins [38], and the iRT sequence (Biognosys). A specific cleavage rule was used for the combination of Lys‐C and trypsin—always cleavage after lysine, and cleavage after arginine, unless proline proceeds. Two missed cleavages were allowed. Carbamidomethylation of cysteine was set as a fixed modification, and protein N‐terminal acetylation and methionine oxidation were set as variable modifications. The Hybrid (DDA + DIA) Library option was selected in DIA Analysis Workflow in Experiment Settings. Data were normalised in Spectronaut using cross‐run local normalisation. No filter type and automatic row selection were applied. Proteins identified by a single stripped peptide sequence (single hit proteins) were excluded from further analysis. Differential protein abundance analysis was performed in Spectronaut Viewer (version 20.3.251119.92449, Biognosys) using an unpaired *t* test. Statistically significant changes of protein abundances were considered for fold‐change ≥1.5 and *Q*‐value ≤0.05. The complete Spectronaut file, together with raw data, has been deposited to the ProteomeXchange Consortium via the PRoteomics IDEntifications (PRIDE) partner repository [39] with the dataset identifier PXD080067.

### 2.15 Transcriptomic and proteomic data integration and concordance analysis

For transcriptomic–proteomic data integration, the proteomic dataset was curated by computationally filtering out common contaminants including bovine proteins and excluding protein groups mapping to multiple gene symbols (e.g., because of shared peptides among homologous proteins, paralogs, or gene families) or corresponding to uncharacterised loci or pseudogenes. The same curation procedure was applied consistently to both the complete proteomic dataset and the subset of differentially abundant proteins.

To characterise the global relationship between the transcriptome and the proteome, transcriptomic and proteomic datasets were merged using unique gene symbols retaining all detected genes and proteins. Concordance was defined as matching directions of RNA and protein LFC values (both positive or both negative) regardless of statistical significance, whereas opposing directions were classified as discordant.

The global concordance percentage was calculated as follows:

$$\text{Concordance}\;\left(\%\right)=\frac{\text{Number of concordant pairs}}{\text{Total co‐detected pairs}}\times 100.$$

To determine if the observed concordance deviated significantly from what would be expected by random chance (50%), a two‐tailed exact binomial test was performed against a null hypothesis (H_0_) of *p* = 0.5. To evaluate the quantitative cross‐platform agreement, a nonparametric Spearman rank correlation (*ρ*) between RNA and protein LFC values was determined. Additionally, a secondary sensitivity analysis was performed using only significantly altered transcripts (FDR < 0.05) with concordance and binomial test statistics recalculated.

### 2.16 Tissue lysate preparation

Tissue lysates were prepared according to a previously established protocol [40]. Briefly, using an Ultra‐Turrax homogenizer (T10; IKA) and homogenisation buffer (2 mM Na_2_HPO_4_, 0.6 mM KH_2_PO_4_, and 22.4 mM NaCl, adjusted to pH 6.0), tissue samples were homogenised on ice. Lysis buffer consisted of 10 mM Tris–HCl (pH 7.5), 1% Triton X‐100, 0.1% SDS, 100 mM NaCl, 1 mM EDTA, 1 mM EGTA, 10% glycerol, and a mixture of protease and phosphatase inhibitors: 25 μM pepstatin A, 200 μM AEBSF, 50 μM E‐64, 5 mM NaF, and 1 mM Na_3_VO_4_. A 1:1 mixture of homogenate and lysis buffer was incubated under agitation for 1 h on a vertical rotator at 4°C and then centrifuged at 22,000*g* for 30 min at 4°C. The supernatant was collected in new microcentrifuge tubes and stored at −80°C.

### 2.17 Protein concentration measurement

The protein concentration was determined using the Pierce™ BCA Protein Assay Kit (Thermo Fisher Scientific), according to the manufacturer's instructions. Absorbance was measured using a 96‐well plate reader (Sunrise; Tecan) at 750 nm.

### 2.18 xMAP multiplex assay

Multiplex immunoanalysis of soluble mediators present in CM was performed using the MILLIPLEX® Human Cytokine/Chemokine/Growth Factor Panel A and Panel B kit (Merck).

In total, 76 analytes were quantified in CM from BrM‐CAF (*n* = 4) and Subgalear‐NF (*n* = 3), prepared as described above. Unconditioned control medium was used as a blank and as a diluent for calibration standards. The immunoassay was performed according to the manufacturer's instructions. Fluorescence intensities were detected using an xMAP Intelliflex DR‐SE version 2.1 according to the manufacturer's instructions. Analyte concentrations were read from 4‐ or 5‐parameter logistic calibration curves constructed in the Belysa analysis software version 1.2.2 (Merck). Only analytes quantifiable in at least three different CM (i.e., above the lower limit of quantification) were included in the further analysis. If a specific sample value was below the limit of detection (LOD), it was substituted for statistical analysis by the LOD divided by the square root of 2 ($\mathrm{LOD}/\sqrt{2}$), as described in [41].

### 2.19 ELISA

Collagen I, fibronectin, and CXCL16 levels in tissue lysates or CM were measured using DuoSet ELISA kits (DuoSet Human Collagen I, Cat. No. DY6220‐05, DuoSet Human Fibronectin, Cat. No. DY1918‐05, and DuoSet Human CXCL16, Cat. No. DY1164, all R&D Systems) following the instructions provided by the manufacturer. FAP protein concentrations were determined in our previously published study [19]. Protein concentrations of collagen I, fibronectin, and FAP were ln‐transformed prior to correlation analysis. Linear association between FAP and collagen I levels was assessed using Pearson's correlation coefficient. Spearman's rank correlation was additionally evaluated on non‐transformed data and yielded consistent results.

### 2.20 Monocyte isolation

Monocytes were isolated from healthy donors' peripheral blood buffy coats. Briefly, Ficoll gradient centrifugation (Ficoll‐buffycoat ratio 3:4, 160*g*, 40 min at RT) was used to separate peripheral blood mononuclear cells. After two washes with PBS and red blood cell lysis (10 min at RT, Red Blood Cell Lysis Solution, Miltenyi), monocytes were separated by negative selection using the Pan‐Monocyte Isolation Kit according to the manufacturer's instructions (Miltenyi). The purity of isolated monocytes was ~95% as verified by flow cytometry analysis of CD14 expression (anti‐CD14, clone CYT‐14F6, Cytognos; 0.125 μL per 50 × 10^3^ cells in 50 μL, 30 min at RT). Monocytes were cultured in RPMI‐1640 + 10% FCS + P/S.

### 2.21 Monocyte migration assay

To evaluate the effect of BrM‐CAF CM on monocyte migration, 2 × 10^5^ monocytes in 100 μL serum‐free RPMI‐1640 were placed in the upper chamber of transwell inserts (24WP, 5.0 μm pores, Corning), and 600 μL of CM or control medium was added to the lower chamber.

To evaluate the role of CCR2/CCL2 signalling, monocytes were pre‐incubated for 30 min with either 1 μM CCR2 antagonist (Merck, Cat. No. 227016) or 0.01% dimethyl sulfoxide (vehicle control; Merck) before seeding into the upper chamber. The lower chamber contained BrM‐CAF CM, control medium, or 100 ng/mL CCL2 (Miltenyi), with all conditions supplemented with either CCR2 antagonist or vehicle control.

In all assays, monocytes were allowed to migrate for 2 h. The upper chamber was then cleaned with a cotton swab. The inserts were fixed with either 10% paraformaldehyde for 10 min followed by staining with 5 μg/mL Hoechst 33258 for 15 min, or with 4% paraformaldehyde (15 min, RT) followed by staining with crystal violet solution (0.05% w/v crystal violet, 1% formalin, and 1% methanol in PBS) for 15 min at RT. For Hoechst‐stained samples, the membrane was washed with PBS, excised using a scalpel blade, and mounted on a microscopic slide with Aqua PolyMount mounting medium (Polysciences). The number of migrating monocytes was evaluated in five visual fields per insert using the 20× objective. The experiments were replicated using monocytes from three different donors.

### 2.22 Immunocytochemistry and immunohistochemistry

Immunofluorescent staining was performed as described previously [19]. Briefly, cells cultured on a coverslip or 10 μm fresh‐frozen tissue sections were fixed using 10% v/v paraformaldehyde in PBS (10 min at RT), permeabilised with 0.1% Triton X‐100 in H_2_O (5 min at RT), and non‐specific antibody binding was blocked with Tris‐buffered saline (TBS) containing 10% FCS and 1% bovine serum albumin (1 h at RT). Primary and secondary antibodies diluted in TBS containing 10% FCS and 1% BSA were used according to Table 1 (immunocytochemistry and analysis of extracellular matrices) and Table 2 (immunohistochemistry). 50 ng/mL Hoechst 33258 (Sigma‐Aldrich) admixed in the secondary antibody solution was used for counterstaining of cell nuclei. After incubation with antibodies, followed by three washes in 0.05% Triton X‐100 in TBS and one wash in H_2_O, the samples were mounted using Aqua PolyMount mounting medium (Polysciences) and visualised using an IX70 fluorescence microscope (Olympus) with an Orca‐flash 4.0 camera (Hamamatsu) or a Stellaris 5 confocal microscope (Leica).

**TABLE 1:** Antibodies used in immunocytochemical analyses and in the analysis of extracellular matrices.

Antigen	Primary antibody clone	Manufacturer	Dilution	Incubation	Secondary antibody^a^	Dilution	Incubation
FAP	F19 (56 μg/mL)	In‐house, from hybridoma culture supernatant	56 μg/mL	Overnight, 4°C	Anti‐Mouse AF488	1:500	1 h, RT
Fibro‐blasts	TE‐7/CBL271	Merck	1:200	Overnight, 4°C	Anti‐Mouse AF488	1:500	1 h, RT
αSMA	1A7	Abcam	1:200	Overnight, 4°C	Anti‐Mouse AF488	1:500	1 h, RT
PDGFRβ	PR7212	LS Bio	1:100	Overnight, 4°C	Anti‐Rabbit AF488	1:500	1 h, RT
Vimentin	VIM‐13.2	Merck	1:200	Overnight, 4°C	Anti‐Mouse AF488	1:500	1 h, RT
Pan‐cyto‐keratin	sAE1/AE3 + 5D3	Abcam	1:200	Overnight, 4°C	Anti‐Mouse AF488	1:500	1 h, RT
GFAP	GF‐01	Exbio	1:200	Overnight, 4°C	Anti‐Mouse AF488	1:500	1 h, RT
CD45	HI30	Exbio	1:200	Overnight, 4°C	Anti‐Mouse AF488	1:500	1 h, RT
Sox2	Polyclonal	Abcam	1:600	Overnight, 4°C	Anti‐Rabbit AF488	1:500	1 h, RT
EpCAM	1B7	Thermo Fisher Scientific	1:200	Overnight, 4°C	Anti‐Mouse AF488	1:500	1 h, RT
Collagen type I	Polyclonal	Abcam	1:800	1 h, RT	Anti‐Rabbit AF488	1:500	1 h, RT
Fibro‐nectin	Polyclonal	Abcam	1:200	1 h, RT	Anti‐Rabbit AF488	1:500	1 h, RT
Ki67	Polyclonal	Abcam	1:200	Overnight, 4°C	Anti‐Rabbit AF488	1:500	1 h, RT

^a^ All secondary antibodies were purchased from Thermo Fisher Scientific.

**TABLE 2:** Antibodies used in immunohistochemical analyses.

Antigen	Primary antibody clone	Manufacturer	Dilution	Incubation	Secondary antibody^a^	Dilution	Incubation
FAP	D8	Vitatex	1:800	Overnight, 4°C	Anti‐Rat AF488	1:500	1 h, RT
CD163	GHI/61	Santa Cruz	1:100	Overnight, 4°C	Anti‐Mouse AF546	1:500	1 h, RT
CD206	15–2	Santa Cruz	1:100	Overnight, 4°C	Anti‐Mouse AF546	1:500	1 h, RT
Collagen type I	Polyclonal	Abcam	1:800	1 h, RT	Anti‐Rabbit AF546	1:500	1 h, RT
Fibro‐nectin	Polyclonal	Abcam	1:200	1 h, RT	Anti‐Rabbit AF546	1:500	1 h, RT

^a^ All secondary antibodies were purchased from Thermo Fisher Scientific.

Immunocytochemical positivity was assessed semi‐quantitatively by visual estimation across the entire sample (approximately 150–200 cells per marker). Cells were considered positive when they exhibited a clear immunofluorescence signal above the background defined by the corresponding negative controls and showed an appropriate staining pattern and morphology. The proportion of positive cells was scored in 5% or 10% increments.

Macrophage proximity to FAP+ BrM‐CAFs was analysed using Fiji (ImageJ). FAP immunopositivity was binarised to define FAP+ regions, which were expanded by 10 μm to generate a “Close to BrM‐CAF” zone; all remaining areas were classified as “Far from BrM‐CAF.” CD163+ or CD206+ macrophages were manually counted in each region.

### 2.23 Statistical analysis

Statistical analyses were performed in GraphPad Prism 8.0.2 (GraphPad Software) and STATISTICA 12 (StatSoft). A two‐sided *p*‐value of <0.05 was considered statistically significant.

In the case of proliferation assays, repeated measures ANOVA was used.

For migration assays, data were analysed using linear mixed‐effects models to account for the hierarchical structure of the experiments and non‐independence of observations. Experimental condition (chemoattractant or treatment) was included as a fixed effect. Random effects accounted for variability between experiments and, for monocyte migration assays, between buffy coat donors. Additional random effects included CM source and transwell insert where applicable, with appropriate nesting of technical replicates within experiments. To satisfy model assumptions, log2 or square‐root transformations of the number of migrating cells were applied where appropriate based on residual diagnostics. Statistical inference was based on pre‐specified contrasts, and *p*‐values were adjusted for multiple testing using the Bonferroni correction.

In other in vitro assays, either the Mann–Whitney *U* test or the Kruskal–Wallis test was applied.

## 3 RESULTS

### 3.1 BrM‐derived fibroblasts exhibit CAF features, senescence, and ECM‐related transcriptional programme

We established several fibroblast‐like cultures (BrM‐CAFs, *n* = 13) from fresh tumour tissue obtained during surgical resection of BrMs of various origins. As controls, normal fibroblasts were isolated from non‐tumorous subgalear connective tissue obtained during BrM surgeries and, in one case, meningioma resection (Subgalear‐NF, *n* = 6). Additionally, we used the same protocol to isolate fibroblast‐like cells from GBM (GBM‐CAF, *n* = 5) and non‐tumorous brain tissue (PRE − PRE‐NF, *n* = 3).

**FIGURE 1:**
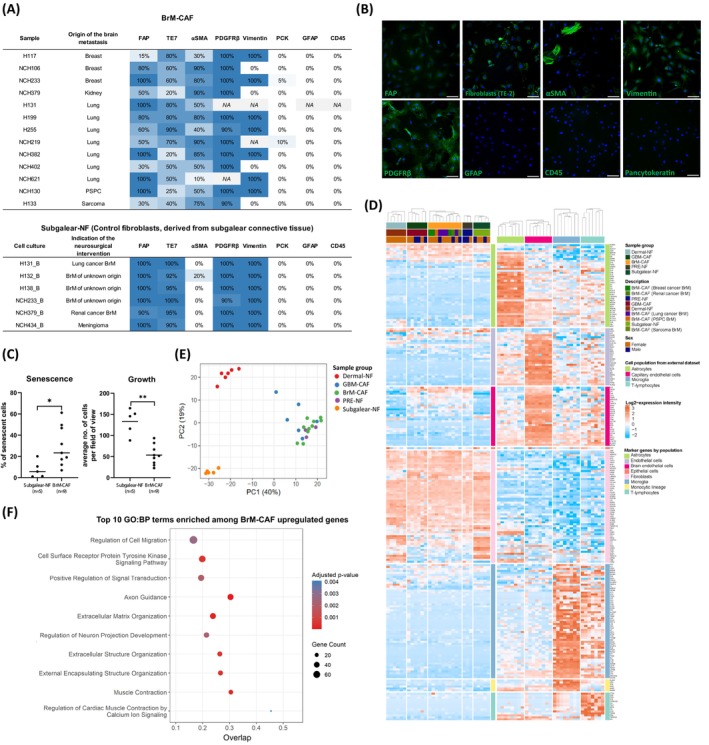
Isolation and characterisation of BrM‐CAFs. (A) Immunocytochemical profiling of mesenchymal (FAP, TE‐7, αSMA, vimentin, PDGFRβ), astroglial (GFAP), immune (CD45) and epithelial cell (PCK) markers in patient‐derived BrM‐CAF and Subgalear‐NF. Values indicate the percentage of immunopositive cells. NA, not analysed; PSPC, primary serous peritoneal carcinoma. (B) Representative images of the immunocytochemical profiling of BrM‐CAF (H255); blue, nuclei; scale bar = 100 μm. (C) Evaluation of BrM‐CAF senescence (determined as the percentage of β‐galactosidase+ cells per visual field), and relative growth (estimated from total cell counts per visual field) within the same images. Each data point represents a distinct BrM‐CAF culture analysed in technical triplicate, averaged from five visual fields per well. *p < 0.05, **p < 0.005, Mann–Whitney test. (D) Heatmap showing relative expression of predefined cell type‐specific marker genes used for lineage identification in patient‐derived fibroblast‐like cultures from BrM and GBM (BrM‐CAF and GBM‐CAF) and in patient‐derived normal fibroblast cultures (Dermal‐NF, Subgalear‐NF, and PRE‐NF). Marker genes were selected based on previously published lineage‐specific signatures. The dataset from [33] was used as a reference for other cell types (astrocytes, endothelial cells, microglia, T lymphocytes). (E) Principal component analysis of patient‐derived fibroblast and fibroblast‐like cultures based on 1000 most variable genes. (F) Overrepresentation analysis of GO:BP terms enriched among genes upregulated in BrM‐CAFs compared with normal fibroblasts (Subgaleal‐NF). To reduce redundancy among significantly enriched GO terms, results were summarised using Revigo based on semantic similarity. GO terms with a dispensability score <0.5 are shown, complete results are provided in Table S4. Dot size indicates gene count and colour indicates adjusted *p*‐value. Overlap represents the fraction of genes annotated to a given GO term that were identified as differentially expressed. Terms on the y‐axis are ordered by gene count. FAP, fibroblast activation protein; αSMA, alpha smooth muscle actin; PDGFRβ, platelet‐derived growth factor receptor type β; GFAP, glial fibrillary acidic protein; PCK, pan‐cytokeratin.

To confirm the mesenchymal characteristics of these cultures, we analysed a panel of mesenchymal and/or CAF markers (FAP, αSMA, PDGFRβ, vimentin), similar to those we previously used for the identification of fibroblast‐like cells in BrM tissues [19]. All BrM‐CAF, GBM‐CAF, PRE‐NF, and Subgalear‐NF cultures exhibited immunopositivity for mesenchymal/CAF markers, with the proportion of positive cells ranging from 10% to 100%. No cells expressing the epithelial marker pan‐cytokeratin, the astrocyte marker GFAP, or the immune cell marker CD45 were detected (Figures 1A,B and S1A). Among the mesenchymal/CAF markers, αSMA was the most informative in discriminating cancer‐associated cells from normal fibroblasts; both BrM‐CAF and GBM‐CAF cultures showed a significantly larger αSMA‐positive subpopulation compared to Subgalear‐NF (Figure S1B).

During in vitro expansion, we noticed a lower proliferation rate of BrM‐CAF cultures compared to normal fibroblasts (Subgalear‐NF). We hypothesised that, similar to CAFs in other tumours, BrM‐CAFs may exhibit features of senescence. Indeed, β‐galactosidase staining revealed a significantly higher proportion of senescent cells in BrM‐CAF cultures compared to Subgalear‐NF (median 23.3% in BrM‐CAF vs. 5.7% in Subgalear‐NF, *p* < 0.05, Mann–Whitney test). Consistent with this observation, cell counts per field of view—used as a proxy for cell growth—were lower in BrM‐CAF cultures (median: 51.6 vs. 145.6 in Subgalear‐NF, *p* < 0.05, Mann–Whitney test; Figures 1C and S2).

To further explore the characteristics of BrM‐CAFs, we performed transcriptomic profiling. To confirm their fibroblast‐like phenotype, we compiled a panel of lineage marker genes characteristic of cell types relevant to the BrM microenvironment (Table S2) based on previously published studies [42, 43, 44, 45, 46]. A heatmap of predefined cell type‐specific marker genes confirmed that BrM‐CAFs, together with other fibroblast cultures, including dermal fibroblasts as a positive control, exhibited a fibroblast lineage signature (Figure 1D). In contrast, markers characteristic of astrocytes, endothelial cells, microglia, and T lymphocytes were largely absent, supporting the fibroblast identity of the analysed cultures and excluding substantial contamination by other major brain‐resident cell populations.

Principal component analysis (PCA) based on 1000 most variable genes revealed that all brain‐derived fibroblast cultures clustered together, with PC1 clearly discriminating them from extracerebral fibroblasts (Subgalear‐NF and Dermal‐NF) (Figures 1E and S1C).

BrM‐CAFs showed pronounced differences compared to normal fibroblasts (Subgalear‐NFs), with 3636 differentially expressed genes (DEGs) identified (Table S3). Overrepresentation analysis using Enrichr (https://maayanlab.cloud/Enrichr/enrich) revealed enrichment of nervous system‐related GO biological process (GO:BP) terms among the upregulated DEGs, including “Axon guidance”. This enrichment corresponded to differential expression of components of the axon guidance‐associated semaphorin–plexin axis and is consistent with the proposed local origin of BrM‐CAFs reported in our previous work [47]. GO analysis of upregulated genes further revealed enrichment of pathways related to extracellular matrix (ECM) organisation and cell migration, including “Positive regulation of cell migration” and “Regulation of cell migration”. Together, these findings indicate that BrM‐CAFs contribute to ECM remodelling and regulation of cell migration within the tumour microenvironment. Furthermore, enrichment of receptor tyrosine kinase signalling and muscle contraction pathways may reflect a signal‐responsive and contractile myofibroblast‐like phenotype of BrM‐CAFs [47] (Figure 1F and Table S4).

In conclusion, we successfully derived fibroblast‐like cells associated with histologically diverse BrMs. These cells exhibited a CAF‐like phenotype, displayed elevated levels of senescence, and their transcriptomic profile suggested a potential role in ECM organisation and regulation of cell migration, pointing to mechanisms through which they may actively shape the metastatic niche.

### 3.2 BrM‐CAFs display deregulated expression of extracellular matrix components and remodelling enzymes

**FIGURE 2:**
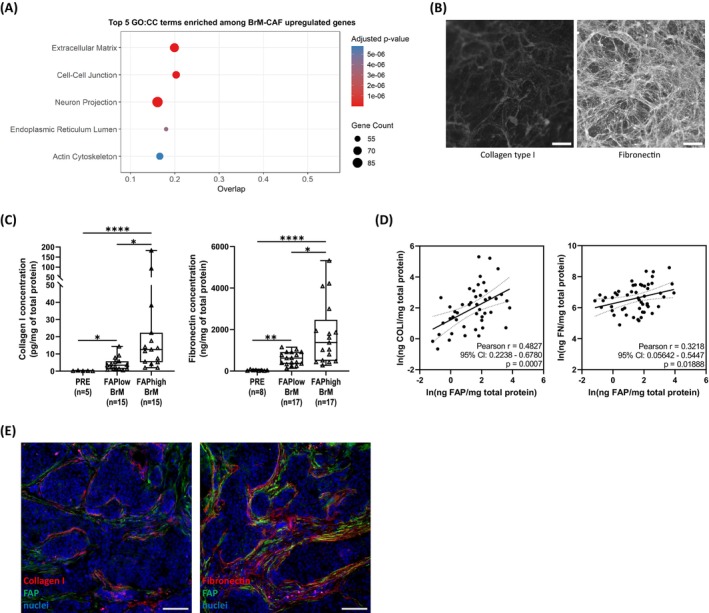
BrM‐CAFs produce extracellular matrix in vitro and are associated with collagen I and fibronectin abundance in BrMs. (A) Overrepresentation analysis of GO:Cellular Component (GO:CC) terms enriched among genes upregulated in BrM‐CAFs compared with normal fibroblasts (Subgaleal‐NF). Dot size indicates gene count and colour indicates adjusted *p*‐value. Overlap represents the fraction of genes annotated to a given GO term that were identified as differentially expressed. To reduce redundancy among significantly enriched GO terms, results were summarised using Revigo based on semantic similarity. GO terms with dispensability <0.1 are shown, complete results are provided in Table S5. Terms on the y‐axis are ordered by adjusted *p*‐value. (B) Immunocytochemical detection of collagen I and fibronectin in extracellular matrices produced by BrM‐CAFs (representative images, BrM‐CAF H131). Scale bar = 100 μm. (C) Collagen I (left panel) and fibronectin (right panel) content in tissue samples of control non‐tumorous brain (PRE) and BrMs stratified according to FAP expression (FAPhigh, upper tercile; FAPlow, lower tercile). Protein concentrations were determined by ELISA and normalised to total protein content. For collagen I, BrMs (FAPhigh FAP > 7.49 ng/mg total protein, *n* = 15; FAPlow FAP < 2.52 ng/mg, *n* = 15), PRE *n* = 5. For fibronectin: FAPhigh FAP > 7.60 ng/mg, *n* = 17; FAPlow FAP < 3.33 ng/mg, *n* = 17; PRE *n* = 8. *p < 0.05, **p < 0.01, ****p < 0.0001, Kruskal–Wallis test. (D) Correlation between FAP levels and collagen I (left; *n* = 46) and fibronectin (right; *n* = 53) concentrations in BrM tissues. Protein concentrations were measured by ELISA and ln‐transformed prior to analysis. Solid lines represent the linear regression fits, and dotted lines indicate the 95% confidence intervals. Corresponding analyses using untransformed data are shown in Figure S4. (E) Representative immunohistochemical images of FAP+ BrM‐CAF and collagen I or fibronectin in BrM tissues (*n* = 5). Scale bar = 50 μm.

“Extracellular Matrix Organisation” was among the most enriched GO:Biological Process terms identified in the transcriptomic analysis, and “Collagen‐Containing Extracellular Matrix” was the top enriched GO:Cellular Component term (Figure 2A and Table S5) highlighting ECM organisation and remodelling as prominent transcriptional features of BrM‐CAFs. This enrichment was driven by upregulated expression of genes encoding collagens (*COL3A1*, *COL4A1*, *COL4A2*, *COL4A3*, *COL4A5*, *COL4A6*, *COL7A1*, *COL8A1*, *COL11A1*, *COL13A1*, *COL18A1*, *COL19A1*, *COL27A1*) and collagen‐modifying enzymes such as lysyl oxidase homologues (*LOXL1*, *LOXL2*, *LOXL3*). Additionally, we identified upregulated expression of ECM remodelling enzymes, notably proteases of the ADAMTS family (*ADAMTS3*, *ADAMTS8*, *ADAMTS12*, *ADAMTS15*, and *ADAMTS18*) and matrix metalloproteinases (*MMP11*, *MMP16*, *MMP25*, and *MMP28*) (Figure S3). To verify the involvement of BrM‐CAFs in the production of ECM, we evaluated their ability to generate fibrillar matrices in vitro using a previously described protocol [25]. Immunocytochemical analysis revealed abundant deposition of fibrillar ECM proteins collagen I and fibronectin (Figure 2B), confirming the matrix production capacity of BrM‐CAFs and supporting their classification as bona fide CAFs, as ECM production is one of the hallmark features of fibroblasts.

To determine whether BrM‐CAF abundance is associated with ECM accumulation in the BrM microenvironment, we quantified collagen I and fibronectin in BrM tissues and non‐tumorous brain samples (Figure 2C). As we previously demonstrated that FAP is expressed in BrM‐CAFs [19], we used FAP levels as a proxy for BrM‐CAF abundance and stratified BrM samples into FAPhigh and FAPlow groups (upper and lower tercile, respectively). Both analysed ECM proteins—collagen I and fibronectin—were markedly enriched in BrMs and their abundance increased with rising FAP expression levels. Compared to the non‐tumorous brain (PRE), BrMs showed substantially higher levels of collagen I (median 7.01 vs. 0.15 ng/mg total protein; *p* < 0.0001, Mann–Whitney test; data not shown) and fibronectin (772.3 vs. 37.8 ng/mg total protein; *p* < 0.0001, Mann–Whitney test; data not shown). Within BrMs, FAPhigh lesions contained higher levels of both proteins than FAPlow lesions—3.66‐fold higher median collagen I and 2.2‐fold higher median fibronectin (both *p* < 0.05, Kruskal–Wallis; Figure 2C). Across BrMs, collagen I and fibronectin levels positively correlated with FAP expression. When analysed as continuous variables, ln‐transformed collagen I concentrations showed a moderate positive correlation with ln‐transformed FAP levels (Pearson *r* = 0.48, 95% confidence interval 0.22–0.68, *p* = 0.0007; *n* = 46; Figure 2D). Similarly, ln‐transformed fibronectin concentrations correlated positively with lnFAP levels (Pearson *r* = 0.32, 95% confidence interval 0.06–0.54, *p* = 0.0188; *n* = 53; Figure 2D). Comparable results were obtained using Spearman rank correlation (Figure S4). Immunohistochemical analysis of BrM tissues (*n* = 5) further confirmed the association of BrM‐CAFs with ECM deposition. Consistent with our previous observations [19], FAP‐positive stromal areas colocalised with both collagen I and fibronectin (Figure 2E).

Together, these findings show that BrM‐CAFs produce fibrillar ECM in vitro and are associated with ECM‐rich microenvironments in human BrM tissues.

### 3.3 BrM‐CAFs express migration‐associated mediators and promote monocyte chemotaxis

**FIGURE 3:**
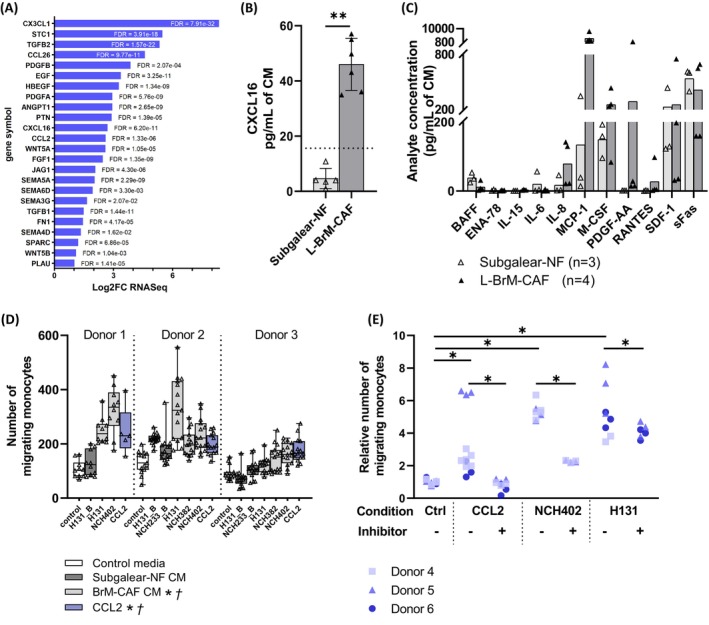
BrM‐CAFs express migration‐associated mediators and promote monocyte migration. (A) Differentially expressed genes between BrM‐CAFs and Subgalear‐NFs associated with the GO Biological Process (GO:BP) terms “Regulation of cell migration” and “Positive regulation of cell migration” encoding proteins with the potential to regulate neighbouring‐cell migration. (B) ELISA quantification of CXCL16 levels in L‐BrM‐CAF (*n* = 6) and Subgalear‐NF (*n* = 5) CM. **p < 0.005, Mann–Whitney test. Bars, mean; error bars, standard deviation; triangles, raw data; dashed line, limit of detection. (C) Multiplex immunoanalysis of L‐BrM‐CAF (*n* = 4) and Subgalear‐NF (*n* = 3) CM using the MILLIPLEX® Cytokine/Chemokine/Growth Factor kit. Analytes detectable in at least three different CM are shown. Bars, mean; triangles, individual CM (mean from technical duplicate). (D) Transwell migration of monocytes toward L‐BrM‐CAF CM, Subgalear‐NF CM, control medium, and recombinant CCL2/MCP‐1, a canonical monocyte chemoattractant (100 ng/mL), used as a positive control. Summary of three experiments, each performed with peripheral monocytes from a different donor in technical triplicate. Triangles represent individual visual fields. *p < 0.05 vs. control medium, ^†^ *p* < 0.05 vs. Subgalear‐NF CM, linear mixed‐effects model. (E) Monocyte transwell migration toward L‐BrM‐CAF CM with CCR2 inhibition. Each data point represents mean from insert. *p < 0.05, linear mixed‐effects model. The number of migrating cells in individual experiments was normalised to the mean number of migrating cells in the corresponding control conditions.

Transcriptomic analysis of BrM‐CAFs revealed increased expression of numerous genes encoding proteins implicated in the regulation of cell migration. Among genes associated with the GO terms “Regulation of cell migration” and “Positive regulation of cell migration,” we identified 24 candidate proteins that could influence migration of neighbouring cells as secreted factors, ECM cues, shed ligands, or membrane‐bound juxtacrine signals. These included multiple chemokines (*CCL2*, *CX3CL1*, *CXCL16*, *CCL26*), growth factors (*PDGFA*, *PDGFB*, *HBEGF*, *EGF*, *FGF1*, *PTN*, *TGFB1*, *TGFB2*, *WNT5A*, *ANGPT1*), matricellular and ECM‐associated proteins (*SPARC*, *FN1*, *PLAU*), and guidance molecules (*SEMA4D*, *SEMA5A*, *SEMA3G*) (Figure 3).

To determine whether these transcriptomic alterations were reflected at the protein level, we performed mass spectrometry analysis of BrM‐CAF cell lysates. Because lung cancer is the most common source of BrMs [1], proteomic analyses were conducted using lung cancer BrM‐CAF cultures (L‐BrM‐CAF) compared with normal fibroblasts (Subgalear‐NFs). Despite the transcriptomic dataset encompassing BrM‐CAFs derived from histologically diverse BrMs, transcriptomic and proteomic fold changes showed substantial agreement across genes and their corresponding protein products quantified in both datasets (*n* = 8503; Spearman *r* = 0.55, *p* < 0.0001; Figure S5A), with 67.8% (*n* = 5761) exhibiting concordant direction of change (two‐tailed binomial test *p* < 0.0001; Figure S5B and Table S6). Of the 24 upregulated candidates identified above, 11 were quantifiable at the protein level by mass spectrometry (Figure S5C). All 11 showed concordant direction of change, and 10 of 11 (91%) were significantly upregulated in L‐BrM‐CAFs. Notably, CX3CL1, which demonstrated the most pronounced upregulation in the transcriptomic analysis, was only quantified in L‐BrM‐CAF lysates while it was undetectable in normal fibroblasts.

We next focused on secreted mediators in CM. Using ELISA, we detected a tenfold higher concentration of CXCL16 in L‐BrM‐CAF CM (*n* = 6) compared with Subgalear‐NF CM (*n* = 5; mean 46 pg/mL vs. 4.6 pg/mL, *p* < 0.005, Mann–Whitney test; Figure 3B). Next, we performed multiplex immunoanalysis using the MILLIPLEX® Cytokine/Chemokine/Growth factor kit to analyse a panel of 76 cytokines in L‐BrM‐CAF (*n* = 4) and Subgalear‐NF (*n* = 3) CM. Eleven cytokines were quantifiable in at least three of the seven CM. Interestingly, both L‐BrM‐CAF and Subgalear‐NF CM contained relatively high levels of SDF‐1/CXCL12. We observed numerically higher levels of IL‐8, IL‐15, MCP‐1/CCL2, M‐CSF, PDGF‐AA, and RANTES in L‐BrM‐CAF CM, whereas IL‐6 and BAFF levels were lower. However, these differences did not reach statistical significance, presumably because of the low sample number and variability between individual cultures (Figure 3C and Table S7).

Given that levels of MCP‐1/CCL2, a key chemokine involved in monocyte recruitment, were more than 11‐fold higher in L‐BrM‐CAF CM than in normal fibroblast CM, we hypothesised that L‐BrM‐CAFs may promote monocyte infiltration into BrMs. Transwell migration assays demonstrated that CM from L‐BrM‐CAFs (*n* = 3) markedly increased monocyte chemotaxis across three donors compared to control media (mean 2.0‐fold, 95% confidence interval 1.6–2.5). Notably, recombinant CCL2 (100 ng/mL) induced a comparable increase in migration (1.9‐fold, 95% confidence interval 1.4–2.6; Figure 3D). To determine whether CCL2 contributes to the pro‐migratory effects of L‐BrM‐CAF CM, we evaluated monocyte migration following pharmacological inhibition of its receptor, CCR2. CM from two independent CAF cultures (H131 and NCH402) consistently increased monocyte migration across three donors, as assessed by linear mixed‐effects modelling (all *p* < 0.001). Pharmacological inhibition of CCR2 markedly reduced monocyte chemotaxis by 54% in NCH402‐induced migration and by 36% in H131‐induced migration (both *p* < 0.001). As expected, CCR2 inhibition exerted a stronger effect on migration induced by recombinant CCL2, reducing chemotaxis by 78% (*p* < 0.001; Figure 3E).

In an IHC analysis of lung BrM tissues (*n* = 5), macrophages were predominantly localised in close proximity to BrM‐CAFs. Quantification revealed that 82% (range 69–95%) of CD163+ macrophages were localised within 10 μm of BrM‐CAFs, whereas only 18% (range 5–31%) were found beyond this distance. A similar distribution was observed for CD206+ macrophages (80%, range 69–92% vs. 20%, range 8–31%). These data indicate preferential localisation of CD163+ and CD206+ macrophages in close proximity to BrM‐CAFs (Figure S6A–C).

Together these findings indicate that BrM‐CAFs constitute a source of diverse migration‐regulating mediators within the metastatic niche. Functional analyses identified CCL2/CCR2 signalling as a contributor to BrM‐CAF‐induced monocyte chemotaxis, and spatial analyses showed that CD163+ and CD206+ macrophages were frequently located near BrM‐CAFs in BrM tissues.

### 3.4 BrM‐CAFs induce CXCL12, CXCL16, and CX3CL1‐dependent migration of cancer cells and promote their invasion, but not growth

Next, we aimed to determine whether L‐BrM‐CAFs enhance the migration and invasion of brain metastatic cancer cells. As a model, we used lung cancer cell lines alongside patient‐derived cancer cells, which offer a more representative system, as metastatic cells constitute a selected and highly adapted population. Primary cancer cell culture (NCH724) was established from a BrM of lung carcinoma and its epithelial phenotype was confirmed by pan‐cytokeratin immunostaining (Figure S7).

**FIGURE 4:**
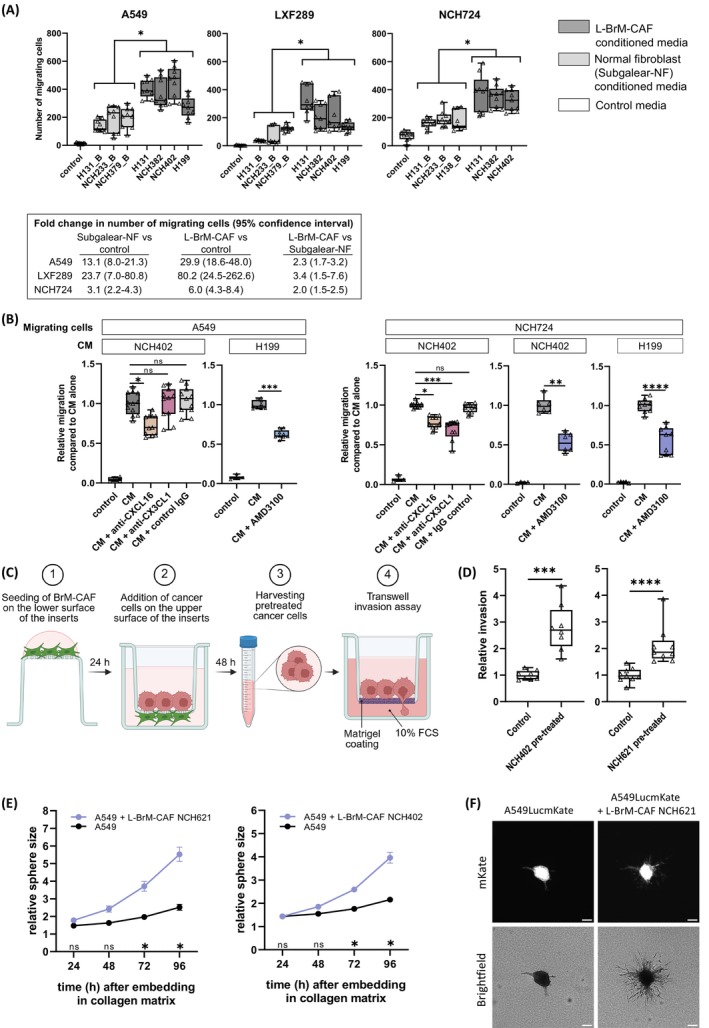
Lung cancer brain metastasis‐associated fibroblasts (L‐BrM‐CAFs) promote cancer cell migration partly dependent on CXCL16, CXCL12, and CX3CL1 and enhance their invasive capacity. (A) Transwell migration of cancer cell lines (A549, LXF289) and L‐BrM patient‐derived cancer cell culture (NCH724) towards CM from L‐BrM‐CAFs or Subgalear‐NF. Triangles—number of migrating cells per visual field (200× magnification, average of five visual fields per insert). *p < 0.05, linear mixed‐effects model. Summary of three experiments, each of which was performed in triplicate. (B) Transwell migration of cancer cells (A549, NCH724) towards CM from L‐BrM‐CAFs (NCH402, H199) with the addition of CXCL16 or CX3CL1 neutralising antibodies (900 ng/mL) or the CXCR4 inhibitor AMD3100 (2.5 μM). Triangles—the number of migrating cells per visual field normalised to CM without inhibitors. **p < 0.01, ***p < 0.001, ****p < 0.0001, Kruskal–Wallis test. Summary of three experiments, each of which was performed in triplicate. (C) Schematic representation of the experimental workflow for indirect coculture of cancer cells and L‐BrM‐CAFs and subsequent transwell invasion assay. (D) Transwell invasion of A549 cancer cells pre‐treated by indirect coculture with L‐BrM‐CAFs (NCH402 or NCH621). ***p < 0.001, ****p < 0.0001, Mann–Whitney test. Summary of three experiments, each of which was performed in triplicate. (E) 3D invasion assay using spheroids composed of cancer cells alone (A549LucmKate) or with admixed L‐BrM‐CAFs (NCH621 or NCH402) embedded in the collagen matrix. For each spheroid, the core area measured 24 h after embedding was used as the reference value for normalisation of all subsequent measurements. Summary of three experiments. ns, not significant; *p < 0.05; repeated measures ANOVA, Tukey post hoc test. The error bars represent SEM. (F) Representative images of spheroids composed of cancer cells alone or with L‐BrM‐CAF 72 h after embedding into a collagen matrix. Scale bar = 200 μm. In (A, B, D), box, 25th to 75th percentile; line, median; whiskers, minimum and maximum values; triangles, raw data.

In a transwell migration assay, L‐BrM‐CAF CM (*n* = 4) markedly increased cancer cell migration compared to control medium (6.0–29.9‐fold) and Subgalear‐NF CM (*n* = 3; 2.0–3.4‐fold) (Figure 4A). We then sought to identify which soluble mediators in the L‐BrM‐CAF CM were responsible for the observed increase in cancer cell migration. Based on transcriptomic profiling and prior studies [20, 48, 49], we identified CXCL16, CX3CL1, and SDF‐1/CXCL12 as candidate mediators. In A549 lung adenocarcinoma cells, migration toward L‐BrM‐CAF CM was reduced by 27% and 38% following treatment with CXCL16‐neutralising antibodies and the CXCL12 receptor inhibitor AMD3100, respectively. In contrast, CX3CL1‐neutralising antibodies had no significant effect (Figure 4B). In patient‐derived NCH724 cells, blocking of the three chemokines decreased migration, with the greatest reduction observed for CXCL12 (mean decrease 47% for CXCL12, 22% for CXCL16, and 32% for CX3CL1) (Figure 4B).

Cancer cell migration and the ability to degrade the ECM are essential for the invasion of tumour cells into surrounding tissues, a hallmark of malignancy. To determine whether L‐BrM‐CAFs influence the invasive behaviour of cancer cells, we first employed an indirect coculture invasion assay. In this system, A549 cells were precultured with L‐BrM‐CAFs (NCH621 or NCH402) using a transwell setup that allows paracrine signalling with limited direct cell–cell contact. Cancer cells precultured with L‐BrM‐CAFs exhibited a significantly increased invasion compared to monocultured controls (2.8 and 2.1‐fold increases in the number of invading cells; Figure 4C,D), indicating that L‐BrM‐CAFs promote a more invasive phenotype in cancer cells. We further validated this finding in a 3D invasion assay. Spheroids composed of fluorescently labelled cancer cells (A549LucmKate) alone or with admixed L‐BrM‐CAFs (NCH621 or NCH402) were embedded in a collagen‐based matrix and the area covered by fluorescent cells was monitored at 24‐h intervals. In spheroids containing L‐BrM‐CAFs, we observed a more pronounced increase in the invasive area over time. At 96 h post‐embedding, the areas covered by CAF‐admixed spheroids were 2.2‐times and 1.8‐fold larger than those of the control A549LucmKate spheroids (Figure 4E,F). Compared with the predominantly collective invasion in cancer‐cell only spheroids, we frequently observed individual, scattered cancer cells emigrating from spheroids containing L‐BrM‐CAFs (Figure S8). Notably, L‐BrM‐CAFs invaded ahead of the cancer cells in the mixed spheroids, suggesting that in addition to inducing a more invasive phenotype in cancer cells, CAF‐associated ECM remodelling may contribute to tumour cell invasion in this model.

Next, we tested the effect of L‐BrM‐CAFs on cancer cell growth. CM from several CAF cultures had no impact on A549 cell growth (data not shown). In direct coculture with A549LucmKate and fluorescently labelled patient‐derived cells (NCH724LucmKate), various L‐BrM‐CAF cultures did not enhance cancer cell growth; instead, cancer cell numbers were modestly lower in several coculture conditions. When cocultured at a 1:1 ratio of L‐BrM‐CAFs to cancer cells, the number of A549LucmKate cells after 72 h was reduced by 21.4% (with NCH621) and 15.6% (with NCH402) compared to A549LucmKate monocultures. Similarly, NCH724LucmKate cell numbers decreased by 10.8% when cocultured with H199. This difference was more pronounced with increasing ratios of L‐BrM‐CAFs to cancer cells (Figure S9A,B). To further characterise the effects of L‐BrM‐CAFs on cancer cell proliferation, we assessed DNA synthesis using an EdU incorporation assay and evaluated expression of the proliferation marker Ki67 in the cancer cell population at a 4:1 L‐BrM‐CAF to cancer‐cell ratio. EdU incorporation assay showed a modest decrease in the proportion of EdU‐positive A549LucmKate cells, from a mean of 44.4% in monoculture to 37.7% and 39.6% in coculture with NCH402 and NCH621, respectively (*p* < 0.01, ANOVA with Dunnet's post hoc test; Figure S9C). In contrast, immunocytochemical analysis of Ki67 revealed a consistently high proportion of Ki67‐positive cancer cells across all conditions (86.7% in monoculture, 94.5% and 93.4% in coculture with NCH402 and NCH621, respectively). However, the overall effect of coculture on Ki67 expression did not reach statistical significance (*p* = 0.063, linear mixed‐effects model) (Figure S9D). Taken together, these findings indicate that L‐BrM‐CAFs exert only limited effects on cancer cell proliferation and do not have a growth‐promoting role under the conditions examined.

**FIGURE 5:**
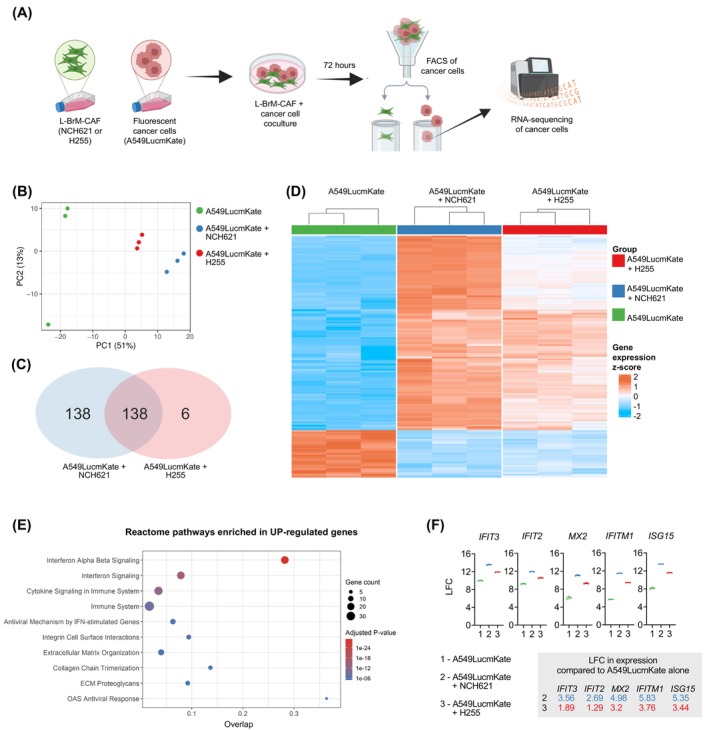
Cancer cell interaction with L‐BrM‐CAFs promotes activation of interferon signalling. (A) Schematic representation of the experimental workflow. A549LucmKate cells were cultured alone or in direct coculture with L‐BrM‐CAFs (NCH621 or H255) for 72 h, followed by FACS of cancer cells and transcriptomic analysis. (B) Principal component analysis of A549LucmKate cells alone or in coculture with L‐BrM‐CAFs (NCH621 or H255) based on 500 most variable genes. (C) Venn diagram showing the number of genes differentially expressed in A549LucmKate cocultured with NCH621 (blue) or H255 (red) compared to A549LucmKate monocultures. (D) Heatmap showing the relative expression of 138 protein‐coding genes consistently differentially expressed in A549LucmKate cocultured with NCH621 or H255. (E) Top 10 Reactome 2024 pathways based on 109 protein‐coding genes consistently upregulated in A549LucmKate cocultured with NCH621 or H255. Overlap represents the fraction of genes annotated to a given GO term that were identified as differentially expressed. Dot size indicates the number of genes contributing to pathway enrichment and colour indicates the adjusted *p*‐value. Complete results are provided in Table S9. (F) Selected IFN signalling pathway genes consistently upregulated in A549LucmKate cocultured with NCH621 or H255. Respective LFC in expression relative to A549LucmKate in monoculture.

To gain insight into the molecular changes induced in cancer cells by interaction with L‐BrM‐CAFs, we cocultured A549LucmKate cells with two different L‐BrM‐CAF cultures (NCH621 or H255) for 3 days, followed by RNA sequencing of cancer cells isolated from the cocultures (Figure 5A). PCA based on the 1000 most variable genes revealed that both L‐BrM‐CAF cultures induced similar expression changes in the transcriptomic profile of A549LucmKate cells after coculture, with more pronounced changes observed in the NCH621 coculture compared to H255 (Figure 5B). We detected 276 differentially expressed protein‐coding genes in A549LucmKate cells after coculture with NCH621 and 144 after coculture with H255. Among these, 138 genes (109 upregulated and 27 downregulated) were consistently altered in both coculture settings (Figure 5C and Table S8). A heatmap based on these 138 differentially expressed genes highlighted the stronger impact of coculture with NCH621 cells (Figure 5D).

Reactome pathway overrepresentation analysis of the upregulated genes identified interferon (IFN) signalling among the most significantly enriched pathways (Figure 5E). While IFN signalling is primarily known for its role in the antiviral response, it has also been implicated in the regulation of cancer cell behaviour. Several IFN‐related genes upregulated in our dataset, including *IFIT3* [50], *IFIT2* [50], and *MX2* [51, 52], have previously been linked to reduced tumour cell proliferation. On the other hand, *IFITM1* [53] and *ISG15* [53] have been associated with increased cancer cell motility and invasion (Figure 5F). Overall, IFN signalling–associated gene expression changes similar to those observed in our study have previously been reported in association with both reduced proliferation and increased invasiveness in cancer cells.

Taken together, our data showed that L‐BrM‐CAFs promoted cancer cell migration, which was mediated at least in part by CXCL16, CX3CL1, and CXCL12. In addition, L‐BrM‐CAFs increased cancer cell invasion in 2D and spheroid assays, while their effects on cancer cell proliferation were limited and did not indicate a growth‐promoting role. Transcriptomic analysis further revealed activation of an IFN signalling‐related gene expression program in cancer cells following coculture with L‐BrM‐CAFs.

## 4 DISCUSSION

The role of stromal cells in BrMs remains poorly understood. In the current study, we provide evidence that BrM‐CAFs contribute to shaping the BrM tumour microenvironment through ECM production and remodelling as well as the secretion of multiple migration‐regulating mediators. Our analyses suggest that BrM‐CAFs may influence both cancer cells and immune cells within the metastatic niche. Transcriptomic profiling indicated a role in the remodelling of the collagen‐containing ECM, which was supported by the ability of BrM‐CAFs to produce abundant fibrillar ECM in vitro and by the association of BrM‐CAF abundance with collagen I and fibronectin levels in patient biopsy specimens. In functional studies, we demonstrated that BrM‐CAF‐derived CCL2 contributed to BrM‐CAF‐induced monocyte chemotaxis, while soluble factors such as CXCL16, CX3CL1, and CXCL12 potently stimulated cancer cell migration. Furthermore, the interaction between BrM‐CAFs and cancer cells increased cancer cell invasion, whereas their effects on cancer cell proliferation were limited and did not indicate a growth‐promoting role.

CAFs have only recently been recognised as integral components of the BrM microenvironment [18, 19, 20, 21]. BrM‐CAF cultures isolated in the current study displayed multiple CAF‐like features and expressed fibroblast lineage marker genes. While BrM‐CAFs differed from fibroblasts of extracerebral origin in their transcriptomic profiles, they showed substantial overlap with both glioblastoma‐associated fibroblasts and fibroblasts from a non‐neoplastic neurological condition (pharmacoresistant epilepsy; PRE‐NFs). The transcriptomic convergence between BrM‐CAFs and PRE‐NFs may reflect two complementary factors: common tissue‐specific pressures and a shared brain‐associated mesenchymal lineage. First, both cell types may be moulded by disease‐related microenvironmental cues within the central nervous system. Pharmacoresistant epilepsy is associated with chronic neuroinflammation [54], and exposure to inflammatory cytokines and chemokines may activate overlapping functional programs in both neoplastic and non‐neoplastic brain fibroblasts, including ECM remodelling. Second, the absence of *HOX* gene expression in both BrM‐CAFs and PRE‐NFs—as we recently reported in Pfeiferová et al. [45]—points to an ectomesenchymal lineage characteristic of brain‐associated mesenchymal cells. Consistent with this interpretation, BrM‐CAFs exhibited increased expression of genes associated with nervous tissue function. The substantial overlap between BrM‐CAFs and PRE‐NFs, together with their clear separation from dermal and subgalear fibroblasts, suggests that brain‐specific mesenchymal identity is a major determinant of global transcriptional programs in these cells. Similarly, Tew et al. also proposed a local origin for the CAF‐like BrM stroma based on the notable concordance of CAF cultures from highly diverse BrM samples [18]. However, CAF cultures isolated from BrMs by Chung et al. were speculated to originate, at least in part, from bone marrow–derived mesenchymal stem cells, based on their ability to transdifferentiate into adipocytes and their expression of the mesenchymal stem cell marker STRO‐1 [20]. This apparent discrepancy may have several explanations. The ectomesenchyme is derived from the neural crest, and neural crest‐derived cells retain a remarkable differentiation potential even in adults [55], potentially mimicking the behaviour of bone marrow mesenchymal stem cells. Alternatively, different experimental approaches may preferentially enrich distinct subsets of the BrM‐CAF population within the resulting cultures. Further research is needed to map the full spectrum of mesenchymal stromal populations in BrMs.

The BrM‐CAF transcriptomic profile pointed to their involvement in ECM remodelling. This is in agreement with previously published single‐cell transcriptomic data from a small cohort of BrMs, which suggested that these cells may contribute to ECM organisation [21]. Our data extend these observations by showing that BrM‐CAFs produce fibrillar ECM in vitro and that FAP expression, used here as a proxy for the abundance of CAFs within BrM tissues, correlates with collagen I and fibronectin protein levels in patient specimens. Together with our previous observation that BrM‐CAFs are localised within collagen‐rich septa in BrMs [19], these tissue‐level data connect the ECM‐enriched transcriptional profile of BrM‐CAFs with collagen I and fibronectin accumulation in human BrM specimens. Furthermore, our recent findings in glioblastoma similarly linked FAP‐positive mesenchymal cells to ECM remodeling [25], suggesting that such stromal programs may represent a common feature of brain tumour microenvironments. Altogether, our findings support an association between FAP‐positive BrM‐CAFs and ECM accumulation. However, the underlying cellular mechanisms remain to be fully dissected. Increased ECM deposition in FAP‐high lesions may arise from direct secretion by BrM‐CAFs, altered ECM turnover and degradation, or activation of ECM‐producing programs in multiple stromal and tumour cell populations by shared microenvironmental cues. In addition, interpretation of FAP‐high lesions requires caution, given that FAP expression has also been reported in other cell populations, including subsets of tumour cells. Future studies employing spatially resolved and single‐cell approaches, together with functional validation in appropriate experimental models, will be required to define the precise cellular sources and regulatory mechanisms shaping ECM landscape within BrMs. In contrast to extracranial solid tumours, where desmoplasia and extensive collagen deposition are generally considered protumorigenic, this phenomenon is not commonly described in brain malignancies, except for several rare, predominantly paediatric tumour types associated with a relatively favourable prognosis [56, 57]. Interestingly, experimentally induced desmoplasia restricted tumour growth in a murine BrM model [18]. Thus, the biological consequences of ECM accumulation in BrMs remain incompletely understood and warrant further investigation.

Different subpopulations of CAFs with specific functional roles in the tumour microenvironment were previously identified in extracranial malignancies and were recently also described in a single‐cell analysis of brain tumours [58]. The myofibroblast‐like CAF (myCAF) subpopulation is characterised by prominent expression of *ACTA2* (encoding αSMA), *COL1*, and *MMP11*, whereas high expression of *IL‐6* and *CXCL12* is characteristic of the inflammatory‐like CAF (iCAF) subpopulation. The BrM‐CAF cultures isolated in our study most closely resemble myCAFs: their αSMA expression was more pronounced compared to normal fibroblasts, and GO:BP analysis revealed an enrichment in muscle contraction‐related pathways. Conversely, secretome analysis did not reveal elevated levels of IL‐6 and CXCL12. Thus, we likely did not isolate iCAFs with our experimental approach; they may have lost their specific characteristics during in vitro culture, or they may have represented only a minor fraction of the CAF population.

This lack of elevated IL‐6 and CXCL12 secretion is intriguing, because both are well‐established components of the so‐called senescence‐associated secretory profile (SASP) [9]. Although we observed a substantial level of senescence in BrM‐CAFs, similar to CAFs in extracranial malignancies, the levels of IL‐6 and CXCL12 were comparable to normal fibroblasts. Instead, we detected higher amounts of IL‐8, CCL2/MCP‐1, both contributing to a pro‐inflammatory milieu, and other SASP components, as well as elevated expression of collagens and TGF‐β. As SASP is subject to substantial spatiotemporal dynamics, we speculate that this may correspond to a specific phase of SASP, such as the Notch‐high phase described by Hoare et al. [55]. Functionally, chemokine secretion by BrM‐CAF may contribute to shaping the immune landscape of BrMs. The expression of CCL2 by BrM‐CAFs, together with increased monocyte chemotaxis toward BrM‐CAF–CM, supports a role for BrM‐associated CAFs in monocyte recruitment. The reduction in monocyte migration following CCR2 inhibition further implicates the CCL2/CCR2 axis in this process. However, the incomplete inhibition suggests that L‐BrM‐CAF‐mediated monocyte chemotaxis is likely a multi‐factorial process involving additional factors. Monocyte‐derived macrophages (MDMs), which arise from circulating monocytes, together with resident microglia, constitute the major myeloid populations in BrMs. These populations display distinct functional phenotypes, with MDMs being enriched in “core matrisome” proteins and thus implicated in ECM remodelling [59]. We observed an accumulation of CD163+ and CD206+ myeloid cells in close proximity to BrM‐CAFs in lung cancer BrMs, mirroring stromal localisation patterns reported in breast cancer BrMs [60]. This spatial association suggests the possibility of BrM‐CAF–myeloid cell crosstalk and raises the possibility that BrM‐CAFs may influence the composition and functional state of the local myeloid compartment, analogous to mechanisms described in extracranial malignancies. In BrMs, MDMs are increasingly recognised as a major myeloid population and frequently exhibit phenotypic features associated with immunosuppressive and tumour‐supportive functions [61]. In this context, BrM‐CAF‐mediated monocyte recruitment may represent one mechanism contributing to the accumulation of these cells within metastatic lesions. Whether BrM‐CAFs also influence the differentiation or functional polarisation of recruited monocytes and macrophages remains to be determined.

In addition to modulating the stromal landscape, increasing evidence indicates that BrM‐CAFs actively influence cancer cell behaviour within the brain metastatic environment. We found that BrM‐CAFs potently attract cancer cells, with chemotaxis mediated at least in part by CXCL12, CXCL16, and CX3CL1, which is in line with previous work in breast cancer BrMs [20]. Together, these observations suggest that BrM‐CAFs may contribute to tumour‐stromal interactions across breast, lung, and potentially other brain metastasis types. In a 3D invasion assay, BrM‐CAFs increased the invasion of tumour cells and BrM‐CAFs were the first to invade the surrounding matrix. This may reflect a CAF‐guided invasion mechanism described in extracranial tumours, where cancer cells preferentially bound to CAF‐produced fibronectin within collagen matrices [62]; as BrM‐CAFs produced fibronectin‐rich matrices in vitro, a similar mechanism could potentially contribute to cancer cell invasion in the BrM context. In addition, cancer cells shifted their invasion mode from collective, protrusion‐like to more frequent dissemination as individual cells, a plasticity that may facilitate cancer cell navigation through the surrounding ECM [63]. Notably, BrM‐CAF “preconditioning” increased the invasiveness of cancer cells even with limited cell‐to‐cell contact, suggesting that BrM‐CAFs induce changes in cancer cells leading to a more invasive phenotype. In contrast to the pronounced pro‐migratory and pro‐invasive effects, we observed only a modest effect on cancer cell proliferation. Rather than promoting cancer cell proliferation, coculture with BrM‐CAFs was associated with a small reduction in cancer cell growth. However, given the limitations inherent to coculture systems, the extent to which this effect reflects direct BrM‐CAF‐mediated regulation rather than indirect consequences of coculture conditions remains uncertain. Transcriptomic profiling of cancer cells exposed to BrM‐CAFs revealed enrichment of interferon signalling–associated gene expression. Previous studies have shown that IFN signalling can influence cancer cell behaviour in a context‐dependent manner. Notably, IFNβ exposure has been reported to reduce proliferation of A549 cells [64] and several IFN‐responsive genes upregulated in cancer cells following coculture with BrM‐CAFs in our study, including *IFITM1*, *ISG1*, have previously been associated with increased invasiveness in other experimental systems [53, 65]. Given the correlative nature of our findings, further functional studies will be required to elucidate the contribution of IFN signalling to the changes in cancer cell behaviour observed in response to BrM‐CAFs.

The contribution of BrM‐CAFs may vary during different stages of BrM progression. Migration and invasion are likely to be particularly relevant during metastatic seeding and early colonisation [66]. However, the BrM‐CAF cultures analysed in this study were derived from established clinical lesions, indicating that pro‐migratory and pro‐invasive CAF‐associated programmes may also be present at later disease stages. Although established BrMs are often regarded as relatively circumscribed lesions, these findings suggest that BrM‐CAFs may continue to influence tumour‐cell behaviour within the evolving metastatic microenvironment throughout disease progression.

From a translational perspective, our findings that BrM‐CAFs remodel the ECM and promote migratory and invasive tumour phenotypes generate testable hypotheses regarding their potential clinical relevance. Although BrMs often appear well‐circumscribed on neuroimaging and during surgical resection, tumour cells may infiltrate the surrounding brain parenchyma in a subset of lesions [67], potentially contributing to local treatment failure. Recently, invasive growth of BrMs has been linked to CHI3L1 release from pSTAT3‐positive astrocytes, highlighting the importance of tumour‐microenvironment interactions in shaping invasive behaviour [68]. While a direct clinical link remains to be established, our in vitro observations raise the possibility that CAF‐enhanced cancer cell motility, combined with CAF‐mediated ECM remodelling, could facilitate local tumour cell dissemination. Future studies integrating BrM‐CAF secretome and ECM signatures with neuroimaging, molecular profiling, and clinical outcome data may help clarify their biological and potential clinical relevance. The frequent FAP expression of BrM‐CAFs also provides opportunities for diagnostic imaging, such as FAPi‐PET [69], and supports further investigation of BrM‐CAFs as a stromal compartment with potential translational relevance. A better understanding of their role within the BrM ecosystem may facilitate the development of future therapeutic strategies, whether aiming to deplete BrM‐CAFs or to modulate their functions.

A limitation of our study is the reliance on in vitro systems. While current murine models of BrMs reproduce several aspects of the brain tumour microenvironment, including neuroinflammatory responses driven by microglia and astroglia [70, 71], they do not fully recapitulate the FAP‐positive mesenchymal stromal compartment and ECM remodelling observed in human BrMs. Consequently, these models may have a limited ability to reproduce the specific BrM‐CAF‐associated processes investigated in the present study. An additional challenge is that CAF activation states are highly dependent on local microenvironmental cues. Even when fibroblast‐like cells are experimentally introduced into animal models, the extent to which they recapitulate and maintain the activated phenotypes observed in human BrM‐CAFs remains uncertain, potentially limiting the biological relevance of such approaches. Future studies using organotypic cultures, patient‐derived ex vivo systems, and other advanced models that better preserve the native stromal compartment and ECM architecture may provide a more physiologically relevant framework for investigating BrM‐CAF function.

Collectively, our findings identify BrM‐CAFs as active stromal components of the brain metastatic niche. They are characterised by ECM‐related transcriptional programmes and fibrillar ECM production, secrete pro‐migratory mediators, promote cancer cell and monocyte migration, and enhance cancer cell invasion. In contrast, their effects on cancer cell proliferation are rather limited and do not indicate a growth‐promoting role, suggesting that BrM‐CAFs may influence metastatic behaviour primarily through microenvironmental remodelling and regulation of migratory and invasive phenotypes rather than through direct stimulation of tumour‐cell growth. These observations support a model in which BrM‐CAFs contribute to the functional organisation of the brain metastatic microenvironment and underscore the importance of this stromal compartment in BrM biology.

## AUTHOR CONTRIBUTIONS

*Conceptualisation*: BV, PB, AS. *Data curation*: LP, BV, HS. *Formal analysis*: BV, LP, TKS, PV, MZ, EB, PB. *Funding acquisition*: AS, PB, BV. *Investigation*: BV, LP, TKS, PV, NT, MZ, JS, SG, EB, JC, HKS. *Methodology*: BV, PV, MZ, EB, MHM, MS, LL, JC. *Project administration*: BV, PB. *Resources*: KS, DN, RT, MK. *Software*: not applicable. *Supervision*: BV, PB. *Validation*: BV, PB, MK, HKS. *Visualisation*: BV, LP. *Writing – original draft*: BV, PB. *Writing – review and editing*: BV, PB, AS, KS.

## FUNDING INFORMATION

This research was funded by the Charles University grant GAUK 342522 and Program Cooperatio, research area “Oncology and Haematology”; project NU22‐03‐00318 of Ministry of Health of the Czech Republic; Ministry of Education, Youth and Sports of the Czech Republic of EATRIS‐CZ LM2023053 and project National Institute for Cancer Research (Programme EXCELES, ID Project No. LX22NPO5102) by the European Union‐Next Generation EU, Project ’Center of Tumor Ecology (CZ.02.1.01/0.0/0.0/16_019/0000785), and the Operational Program Research, Development and Education.

## CONFLICT OF INTEREST STATEMENT

The authors declare the following financial interests/personal relationships which may be considered as potential competing interests: Petr Busek reports financial support was provided by Ministry of Health of the Czech Republic. Aleksi Sedo reports financial support was provided by Ministry of Education Youth and Sports of the Czech Republic. If there are other authors, they declare that they have no known competing financial interests or personal relationships that could have appeared to influence the work reported in this paper.

## Supporting information


**Table S1.** Clinical characteristics of patients, origin of brain metastases (when available), and analyses performed on individual samples.

**Table S2.** Lineage marker genes characteristic of fibroblasts and other cell types relevant to the brain metastasis microenvironment.

**Table S3.** Differentially expressed genes between BrM‐CAFs and Subgalear‐NFs.

**Table S4.** Complete Gene Ontology Biological Process (GO:BP) overrepresentation analysis results for genes upregulated in BrM‐CAFs compared with Subgalear‐NFs.

**Table S5.** Complete Gene Ontology Cellular Component (GO:CC) overrepresentation analysis results for genes upregulated in BrM‐CAFs compared with Subgaleal‐NFs.

**Table S6.** Concordance analysis of transcriptomic and proteomic expression changes in BrM‐CAFs in comparison to Subgalear‐NFs.

**Table S7.** Results of the multiplex immunoanalysis of conditioned media from L‐BrM‐CAFs (*n* = 4) and Subgalear‐NFs (*n* = 3) using the MILLIPLEX® Cytokine/Chemokine/Growth factor kit.

**Table S8.** Differentially expressed genes between A549LucmKate cultured alone or with the addition of BrM‐CAFs. Genes differentially expressed in both co‐culture conditions are highlighted in yellow.

**Table S9.** Complete results of Reactome pathway overrepresentation analysis for genes upregulated in A549LucmKate cells co‐cultured with BrM‐CAFs compared to A549LucmKate cells cultured alone.

**Figure S1.** Immunocytochemical and transcriptomic profiling of fibroblast cultures from diverse origins. (A) Immunocytochemical profiling of mesenchymal (FAP, TE‐7, αSMA, Vimentin, PDGFRβ), astroglial (GFAP), immune (CD45), and epithelial cell (pan‐cytokeratin = PCK) marker expression in patient‐derived fibroblast‐like cultures from subgalear connective tissue (Subgalear‐NF), pharmacoresistant epilepsy (PRE‐NF), and glioblastoma (GBM‐CAF). Values indicate the percentage of immunopositive cells; NA, not analysed. (B) Percentage of αSMA‐positive cells determined by immunocytochemistry. Each data point represents an individual cell culture. **p < 0.01, Kruskal–Wallis test. (C) Heatmap depicting relative expression of 1000 most variable genes among analysed fibroblast cultures (BrM‐CAF, Subgalear‐NF, GBM‐CAF, PRE‐NF, Dermal‐NF).

**Figure S2.** Representative senescence‐associated β‐galactosidase staining of brain metastasis‐associated fibroblasts (BrM‐CAFs) and control subgalear normal fibroblasts (Subgalear‐NFs).
Blue staining indicates senescent cells. Corresponding phase‐contrast and bright‐field images are shown. Scale bar = 50 μm.

**Figure S3.** GO:BP Extracellular matrix organisation: deregulated genes in BrM‐CAFs. Differentially expressed genes between BrM‐CAFs and Subgalear‐NFs included in the GO:BP term Extracellular matrix organisation.

**Figure S4.** Correlation between FAP protein concentration and collagen I and fibronectin in brain metastasis tissues. Scatter plots showing the relationship between FAP and collagen I (left; *n* = 46) and fibronectin (right; *n* = 53) concentrations. Protein concentrations were measured by ELISA. Spearman correlation coefficients, 95% confidence intervals, and *p*‐values are indicated.

**Figure S5.** Concordance between transcriptomic and cell‐lysate proteomic fold‐changes in BrM‐CAF compared with Subgalear‐NF controls. (A) Relationship between transcriptomic and proteomic LFC for genes quantified in both datasets. Each point represents a matched transcript‐protein pair. The x‐axis shows RNASeq LFC and the y‐axis shows proteomic LFC. Vertical and horizontal lines indicate zero‐fold change. All quantified transcript‐protein pairs are shown in black (*n* = 8503; Spearman *ρ* = 0.55, *p* < 0.0001), genes significantly differentially expressed in RNASeq (FDR < 0.05) are shown in brown (*n* = 3976; Spearman *ρ* = 0.71, *p* < 0.0001). (B) Directional concordance between transcriptomic and proteomic changes. Transcript‐protein pairs were classified as concordant when RNASeq and proteomic fold changes had the same direction (both increased or both decreased) and discordant when directions differed. Among all quantified pairs, 67.8% (5761/8503) showed concordant direction of change, significantly greater than expected by chance (binomial test, *p* < 0.0001). Concordance increased to 78.8% for genes significantly differentially expressed in RNASeq (FDR < 0.05; data not shown). (C) Migration‐associated genes upregulated in BrM‐CAFs with corresponding proteomic validation. Differentially expressed genes in BrM‐CAFs relative to Subgalear‐NF control fibroblasts associated with the GO terms “Regulation of cell migration” and “Positive regulation of cell migration” are shown. Genes are ordered according to RNA‐seq LFC. Corresponding proteomic measurements from cell‐lysate mass spectrometry are shown where available and include average log_2_ protein ratios, *q*‐values, and statistical significance (*q < 0.05). Concordance indicates agreement in the direction of change between transcriptomic and proteomic datasets. ^#^CX3CL1 was detected exclusively in BrM‐CAFs by mass spectrometry and was therefore not included in the proteomic differential expression analysis.

**Figure S6.** Spatial proximity of BrM‐CAFs and tumour‐associated macrophages in lung cancer brain metastases. (A) Representative IHC images of FAP+ BrM‐CAFs and tumour‐associated macrophages expressing the macrophage markers CD163 or CD206 in lung cancer BrM tissue (*n* = 5). Scale bar = 100 μm. (B) Quantification of the proportion of tumour‐associated macrophages located close to BrM‐CAF (<10 μm) vs. distant from FAP+ BrM‐CAF (>10 μm) in lung cancer BrM tissue (*n* = 5). *p < 0.05, paired *t* test. (C) Representative IHC image showing the 10 μm distance threshold used for spatial analysis of macrophage localisation relative to FAP+ BrM‐CAFs. White outlines delineate the 10 μm region surrounding FAP+ cells.

**Figure S7.** Immunocytochemical profiling of patient‐derived cancer cell culture NCH724. Immunocytochemical profiling of mesenchymal (FAP, TE‐7, αSMA, Vimentin, PDGFRβ), astroglial (GFAP), immune cell (CD45) and epithelial cell (pan‐cytokeratin = PCK) marker expression in BrM patient‐derived cancer cell culture NCH724 with representative photos. Values indicate the percentage of immunopositive cells. Blue, nuclei; scale bar = 100 μm.

**Figure S8.** 3D spheroid invasion assay: representative images. Representative images of spheroids composed of fluorescent cancer cells (A549LucmKate) alone or with admixed BrM‐CAF (NCH621 or NCH402). Photos were acquired every 24 h, starting at 24 h after embedding the spheroid in collagen‐based matrix. Insets highlight the invasive edge of the spheroids, showing protrusions in cancer cell spheroids and individual invading cells in spheroids containing BrM‐CAFs. Scale bar = 200 μm.

**Figure S9.** Cancer cell proliferation in direct coculture with L‐BrM‐CAFs. (A) Live‐cell longitudinal cell counts. A549LucmKate or NCH724LucmKate cancer cells (CC) were cultured alone or in direct coculture with L‐BrM‐CAFs (NCH621, NCH402, or H199) at ratios of 4:1, 1:1, or 1:4. Cell numbers were monitored via automated imaging at 8‐h intervals over 72 h. Summary data from three independent experiments are shown. *p < 0.05 compared with monoculture (repeated measures ANOVA, Tukey's post hoc test). (B) Representative images from longitudinal cell tracking. Micrographs show A549LucmKate cancer cells in direct coculture with NCH621 CAFs at a 4:1 (CAF:CC) ratio after 72 h. Individual panels display the bright‐field view (left) and mKate fluorescence (right). Scale bar = 300 μm. (C) EdU incorporation assay. Percentage of EdU‐positive A549LucmKate cells cultured alone or in coculture with NCH402 or NCH621 at a 4:1 (CAF:CC) ratio for 48 h. Points represent individual biological replicates (*n* = 4). Horizontal bars indicate mean values ± SD. **p < 0.01, ANOVA with Dunnet's post hoc test. (D) Ki67 immunocytochemical staining. Percentage of Ki67‐positive cancer cells after 72 h in monoculture or coculture with NCH402 or NCH621 at a 4:1 (CAF:CC) ratio. Horizontal bars indicate the slide mean ± SD; for each slide (*n* = 3 per condition), the mean was calculated from five independent fields of view. The overall effect of coculture did not reach statistical significance (*p* = 0.063, linear mixed‐effects model).

## Data Availability

The transcriptomic datasets used in this article are available in the ArrayExpress database (https://www.ebi.ac.uk/biostudies/arrayexpress) under the accession numbers E‐MTAB‐13242 and E‐MTAB‐15451. The proteomic datasets, including complete Spectronaut file, together with raw data, have been deposited to the ProteomeXchange Consortium via the PRoteomics IDEntifications (PRIDE) partner repository with the dataset identifier PXD080067.
