# Supplementary material for: Brain metastasis‐associated fibroblasts shape the tumour microenvironment to enhance cancer cell invasion
Source: Brain Pathol. 2026 Aug 4:e70129. Online ahead of print. doi: 10.1111/bpa.70129 (PMC13437050; doi:10.1111/bpa.70129)
Supplement: Supplementary file 1 [file BPA-9999-e70129-s001.pdf]

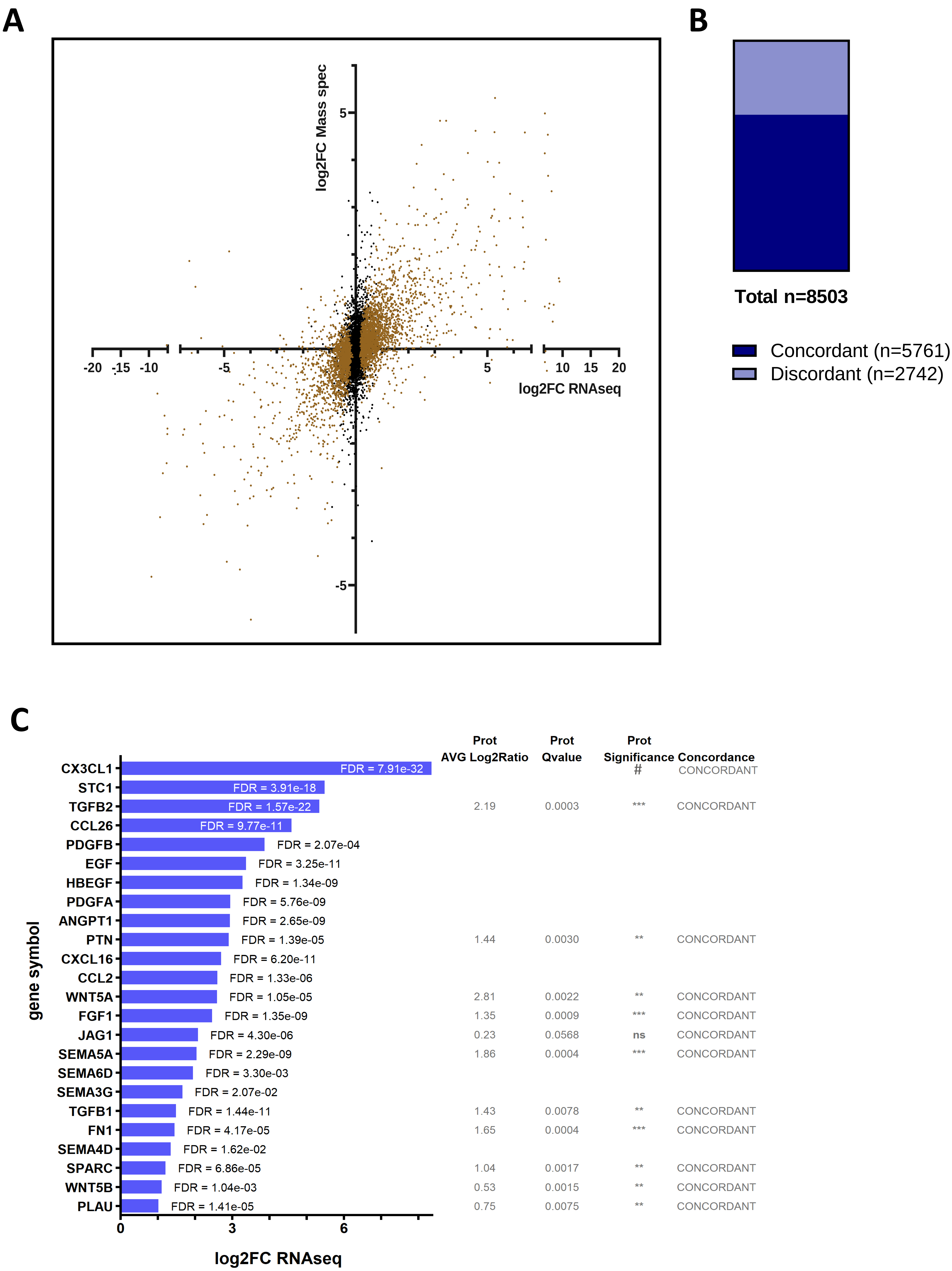
A
log2FC Mass spec
5
-5
-20 -15 -10 -5 5 10 15 20
log2FC RNAseq
B
Total n=8503
Concordant (n=5761)
Discordant (n=2742)
C
gene symbol
log2FC RNAseq
0 3 6
Prot AVG Log2Ratio
Prot Qvalue
Prot Significance
Concordance
CX3CL1 FDR = 7.91e-32 # CONCORDANT
STC1 FDR = 3.91e-18
TGFB2 FDR = 1.57e-22 2.19 0.0003 *** CONCORDANT
CCL26 FDR = 9.77e-11
PDGFB FDR = 2.07e-04
EGF FDR = 3.25e-11
HBEGF FDR = 1.34e-09
PDGFA FDR = 5.76e-09
ANGPT1 FDR = 2.65e-09
PTN FDR = 1.39e-05 1.44 0.0030 ** CONCORDANT
CXCL16 FDR = 6.20e-11
CCL2 FDR = 1.33e-06
WNT5A FDR = 1.05e-05 2.81 0.0022 ** CONCORDANT
FGF1 FDR = 1.35e-09 1.35 0.0009 *** CONCORDANT
JAG1 FDR = 4.30e-06 0.23 0.0568 ns CONCORDANT
SEMA5A FDR = 2.29e-09 1.86 0.0004 *** CONCORDANT
SEMA6D FDR = 3.30e-03
SEMA3G FDR = 2.07e-02
TGFB1 FDR = 1.44e-11 1.43 0.0078 ** CONCORDANT
FN1 FDR = 4.17e-05 1.65 0.0004 *** CONCORDANT
SEMA4D FDR = 1.62e-02
SPARC FDR = 6.86e-05 1.04 0.0017 ** CONCORDANT
WNT5B FDR = 1.04e-03 0.53 0.0015 ** CONCORDANT
PLAU FDR = 1.41e-05 0.75 0.0075 ** CONCORDANT
