# Supplementary material for: Brain metastasis‐associated fibroblasts shape the tumour microenvironment to enhance cancer cell invasion
Source: Brain Pathol. 2026 Aug 4:e70129. Online ahead of print. doi: 10.1111/bpa.70129 (PMC13437050; doi:10.1111/bpa.70129)
Supplement: Supplementary file 2 [file BPA-9999-e70129-s003.pdf]

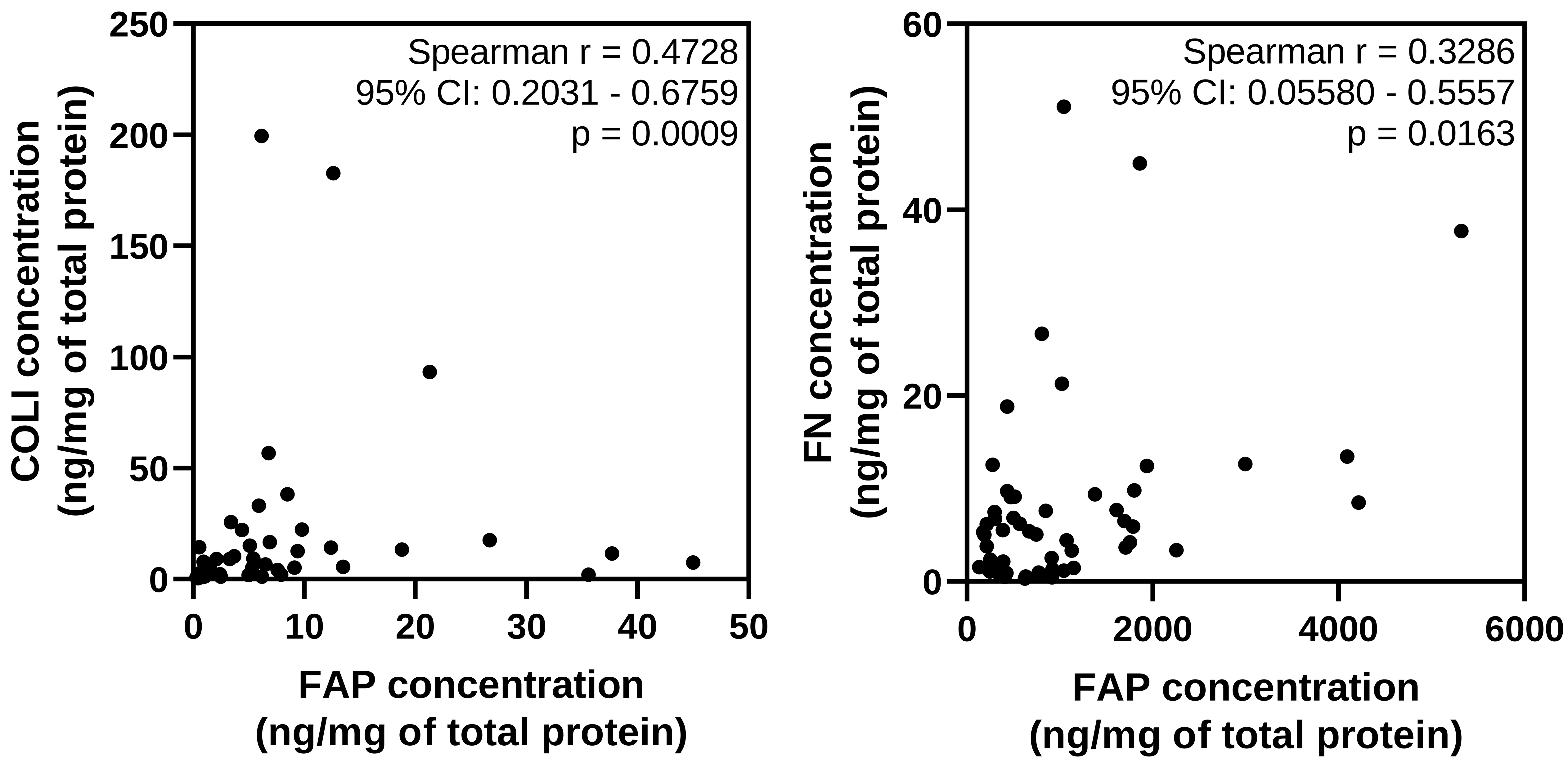
Spearman r = 0.4728
95% CI: 0.2031 - 0.6759
p = 0.0009
COLI concentration
(ng/mg of total protein)
FAP concentration
(ng/mg of total protein)
Spearman r = 0.3286
95% CI: 0.05580 - 0.5557
p = 0.0163
FN concentration
(ng/mg of total protein)
FAP concentration
(ng/mg of total protein)
