# Supplementary material for: Brain metastasis‐associated fibroblasts shape the tumour microenvironment to enhance cancer cell invasion
Source: Brain Pathol. 2026 Aug 4:e70129. Online ahead of print. doi: 10.1111/bpa.70129 (PMC13437050; doi:10.1111/bpa.70129)
Supplement: Supplementary file 3 [file BPA-9999-e70129-s004.pdf]

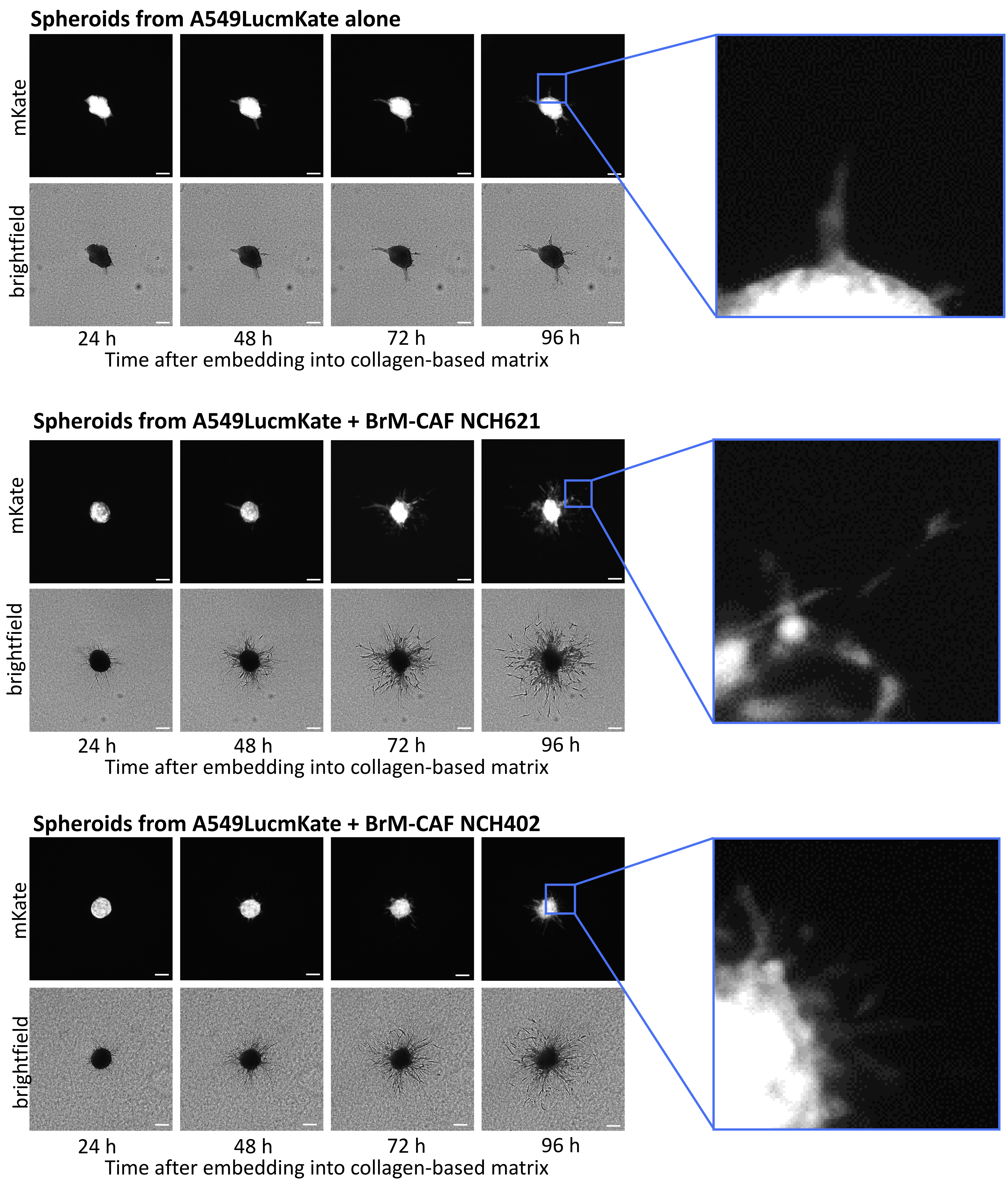
Spheroids from A549LucmKate alone
mKate
brightfield
24 h
48 h
72 h
96 h
Time after embedding into collagen-based matrix
Spheroids from A549LucmKate + BrM-CAF NCH621
mKate
brightfield
24 h
48 h
72 h
96 h
Time after embedding into collagen-based matrix
Spheroids from A549LucmKate + BrM-CAF NCH402
mKate
brightfield
24 h
48 h
72 h
96 h
Time after embedding into collagen-based matrix
