# Supplementary material for: Brain metastasis‐associated fibroblasts shape the tumour microenvironment to enhance cancer cell invasion
Source: Brain Pathol. 2026 Aug 4:e70129. Online ahead of print. doi: 10.1111/bpa.70129 (PMC13437050; doi:10.1111/bpa.70129)
Supplement: Supplementary file 4 [file BPA-9999-e70129-s005.pdf]

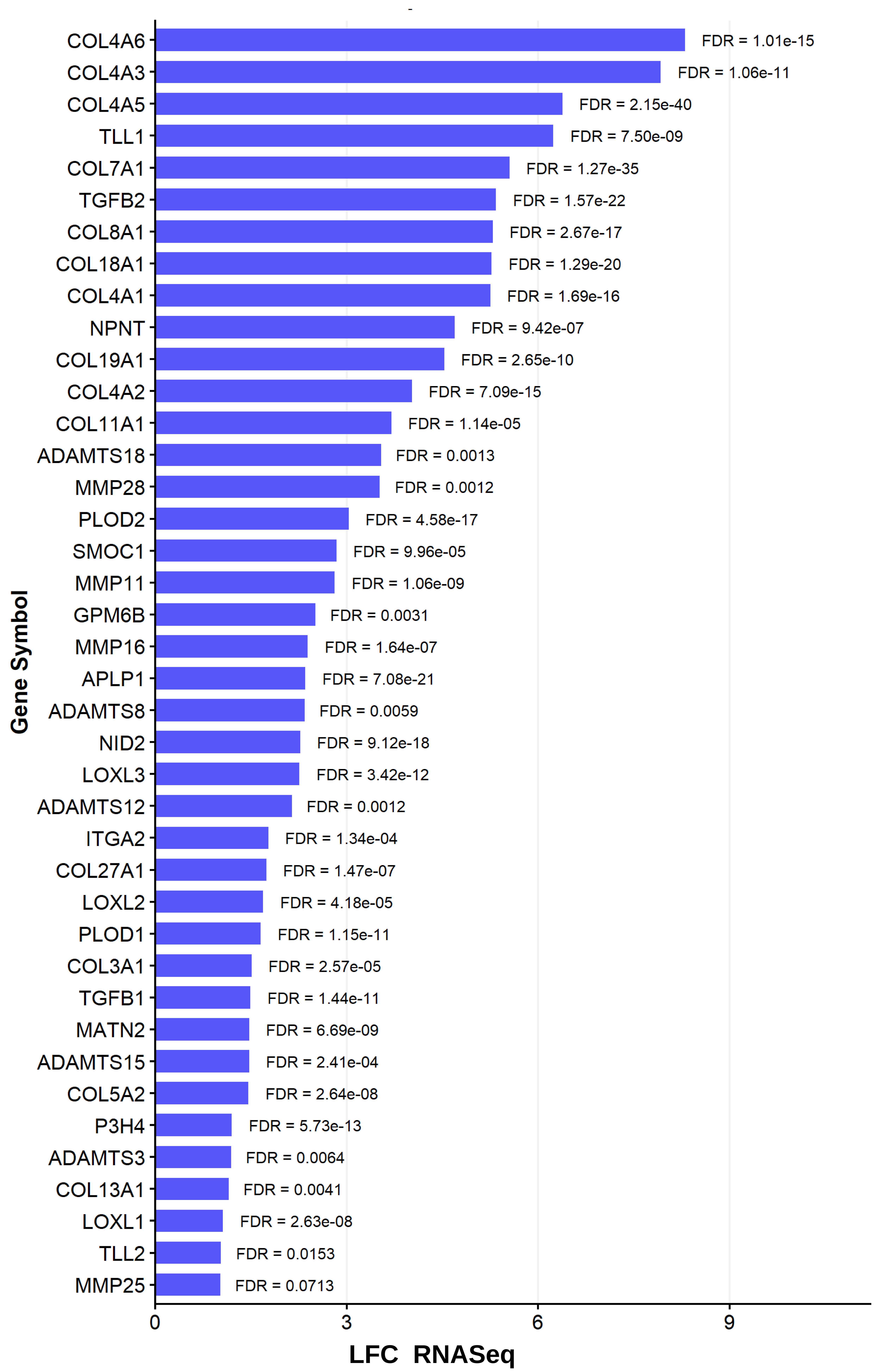
COL4A6
COL4A3
COL4A5
TLL1
COL7A1
TGFB2
COL8A1
COL18A1
COL4A1
NPNT
COL19A1
COL4A2
COL11A1
ADAMTS18
MMP28
PLOD2
SMOC1
MMP11
GPM6B
MMP16
APLP1
ADAMTS8
NID2
LOXL3
ADAMTS12
ITGA2
COL27A1
LOXL2
PLOD1
COL3A1
TGFB1
MATN2
ADAMTS15
COL5A2
P3H4
ADAMTS3
COL13A1
LOXL1
TLL2
MMP25
FDR = 1.01e-15
FDR = 1.06e-11
FDR = 2.15e-40
FDR = 7.50e-09
FDR = 1.27e-35
FDR = 1.57e-22
FDR = 2.67e-17
FDR = 1.29e-20
FDR = 1.69e-16
FDR = 9.42e-07
FDR = 2.65e-10
FDR = 7.09e-15
FDR = 1.14e-05
FDR = 0.0013
FDR = 0.0012
FDR = 4.58e-17
FDR = 9.96e-05
FDR = 1.06e-09
FDR = 0.0031
FDR = 1.64e-07
FDR = 7.08e-21
FDR = 0.0059
FDR = 9.12e-18
FDR = 3.42e-12
FDR = 0.0012
FDR = 1.34e-04
FDR = 1.47e-07
FDR = 4.18e-05
FDR = 1.15e-11
FDR = 2.57e-05
FDR = 1.44e-11
FDR = 6.69e-09
FDR = 2.41e-04
FDR = 2.64e-08
FDR = 5.73e-13
FDR = 0.0064
FDR = 0.0041
FDR = 2.63e-08
FDR = 0.0153
FDR = 0.0713
Gene Symbol
0
3
6
9
LFC RNASeq
