# Supplementary material for: Brain metastasis‐associated fibroblasts shape the tumour microenvironment to enhance cancer cell invasion
Source: Brain Pathol. 2026 Aug 4:e70129. Online ahead of print. doi: 10.1111/bpa.70129 (PMC13437050; doi:10.1111/bpa.70129)

A

PRE-NF (Fibroblasts derived from non-malignant brain tissue)

Cell culture	Indication of the neurosurgical intervention	FAP	TE7	αSMA	PDGFRβ	Vimentin	PCK	GFAP	CD45
H158_F	Pharmacoresistant epilepsy	10%	50%	10%	50%	0%	*NA*	0%	0%
NCH236_F	Pharmacoresistant epilepsy	100%	95%	50%	100%	100%	*NA*	0%	0%
NCH421_F	Pharmacoresistant epilepsy	0%	90%	0%	100%	0%	*NA*	0%	0%

GBM-CAF (Glioblastoma derived fibroblasts)

Cell culture	Indication of the neurosurgical intervention	FAP	TE7	αSMA	PDGFRβ	Vimentin	PCK	GFAP	CD45
NCH197G	Glioblastoma	100%	20%	95%	100%	100%	*NA*	0%	0%
NCH230G	Glioblastoma	70%	50%	30%	95%	0%	*NA*	0%	0%
NCH347G	Glioblastoma	80%	20%	60%	70%	0%	*NA*	0%	0%
NCH348G	Glioblastoma	100%	75%	95%	100%	100%	*NA*	0%	0%
NCH353G	Glioblastoma	50%	50%	55%	100%	50%	*NA*	0%	0%
NCH413G	Glioblastoma	90%	20%	90%	100%	100%	0%	0%	5%

B

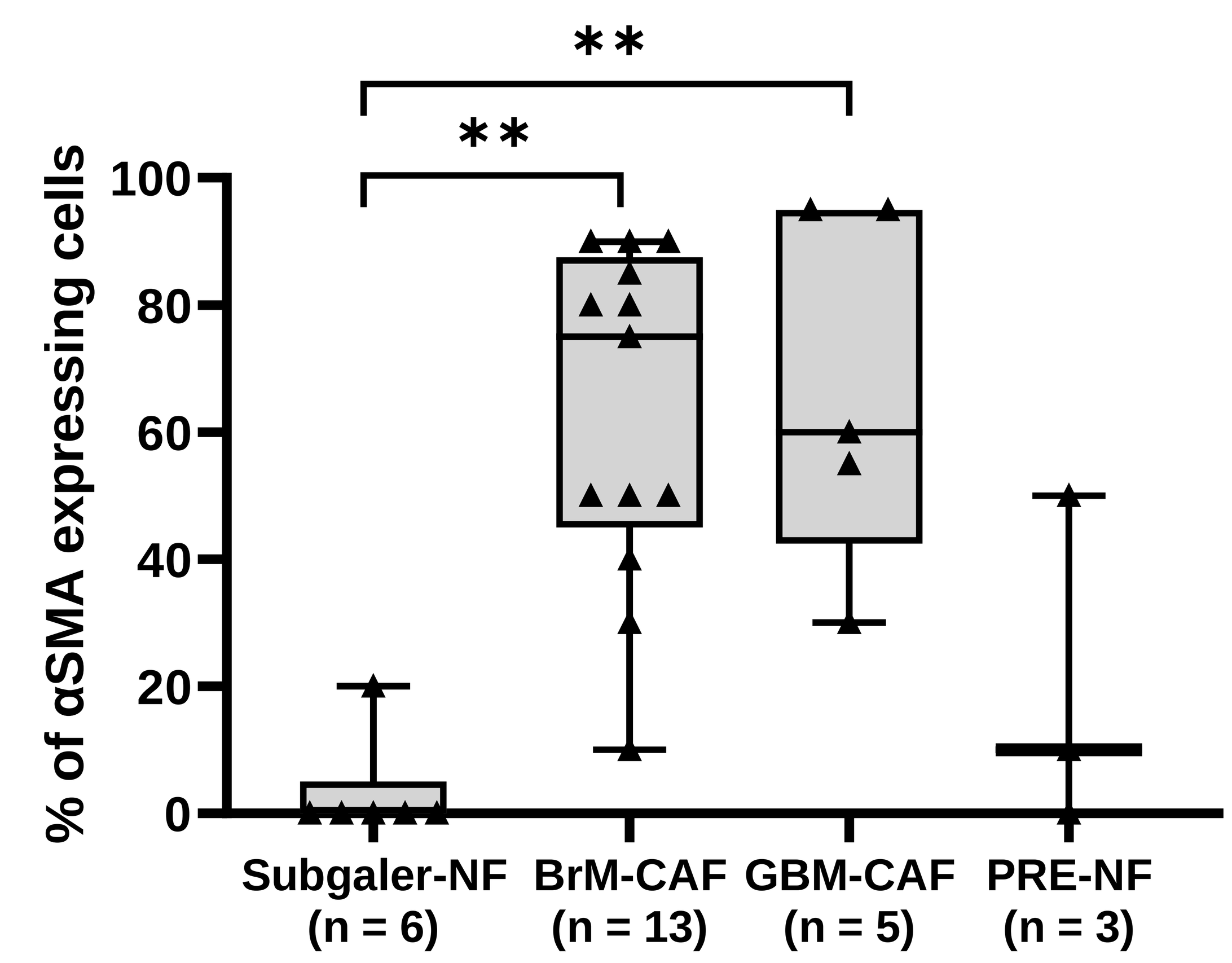

C

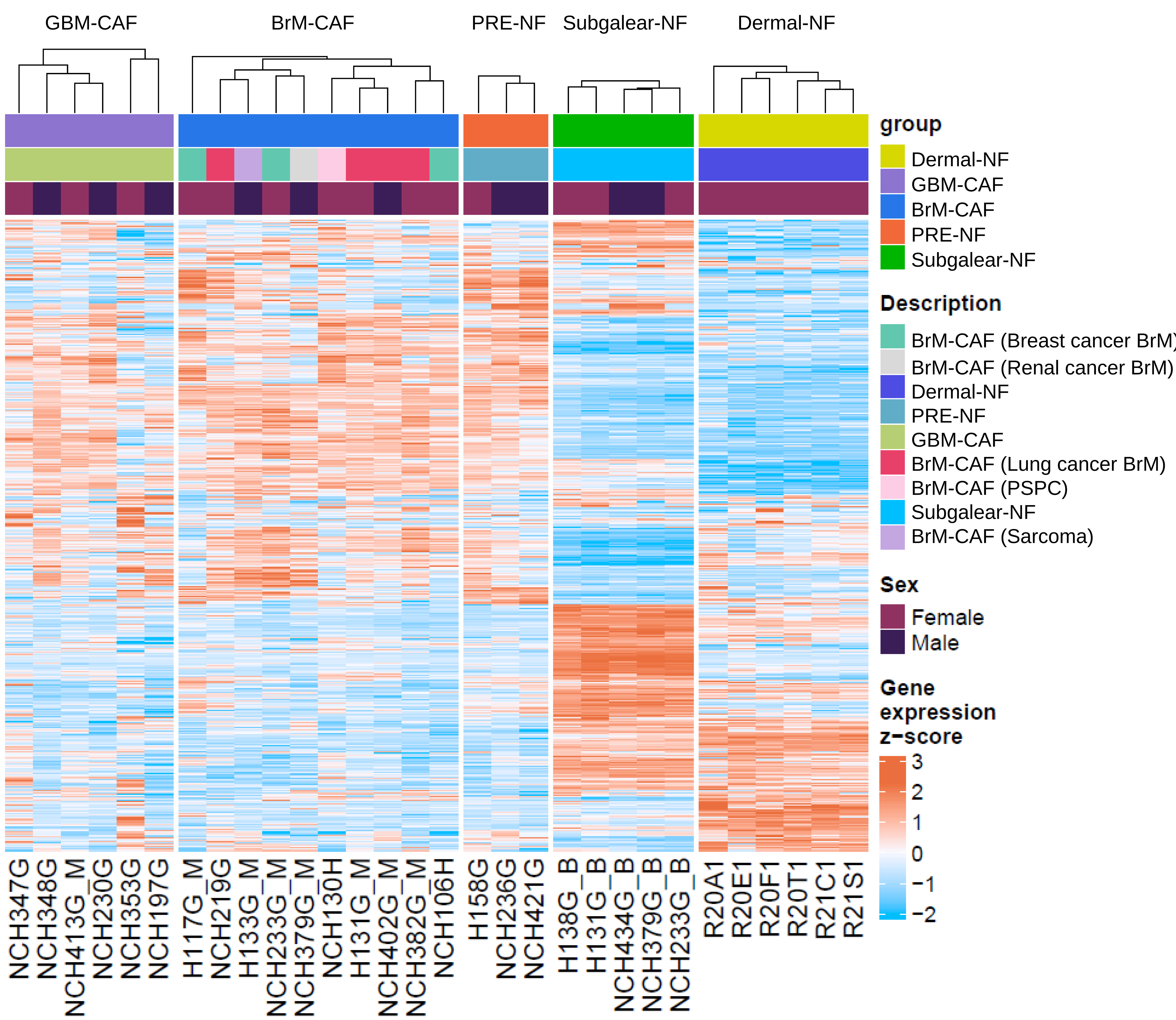

Supplement: Supplementary file 5 [file BPA-9999-e70129-s008.pdf]
