# Supplementary material for: Brain metastasis‐associated fibroblasts shape the tumour microenvironment to enhance cancer cell invasion
Source: Brain Pathol. 2026 Aug 4:e70129. Online ahead of print. doi: 10.1111/bpa.70129 (PMC13437050; doi:10.1111/bpa.70129)
Supplement: Supplementary file 6 [file BPA-9999-e70129-s011.pdf]

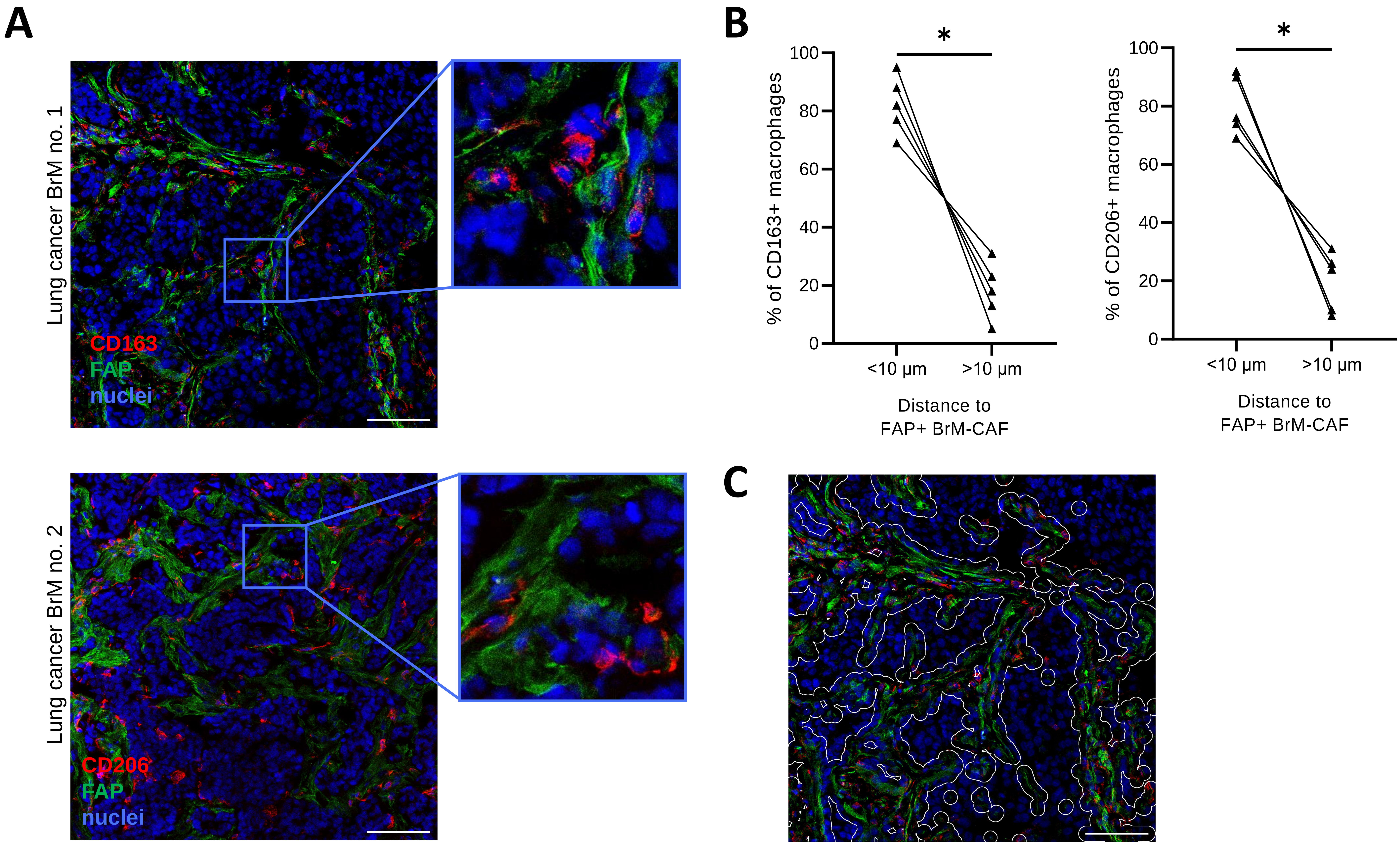
A
Lung cancer BrM no. 1
CD163
FAP
nuclei
Lung cancer BrM no. 2
CD206
FAP
nuclei
B
*
% of CD163+ macrophages
100
80
60
40
20
0
<10 µm
>10 µm
Distance to
FAP+ BrM-CAF
*
% of CD206+ macrophages
100
80
60
40
20
0
<10 µm
>10 µm
Distance to
FAP+ BrM-CAF
C
