# Supplementary material for: Brain metastasis‐associated fibroblasts shape the tumour microenvironment to enhance cancer cell invasion
Source: Brain Pathol. 2026 Aug 4:e70129. Online ahead of print. doi: 10.1111/bpa.70129 (PMC13437050; doi:10.1111/bpa.70129)
Supplement: Supplementary file 7 [file BPA-9999-e70129-s013.pdf]

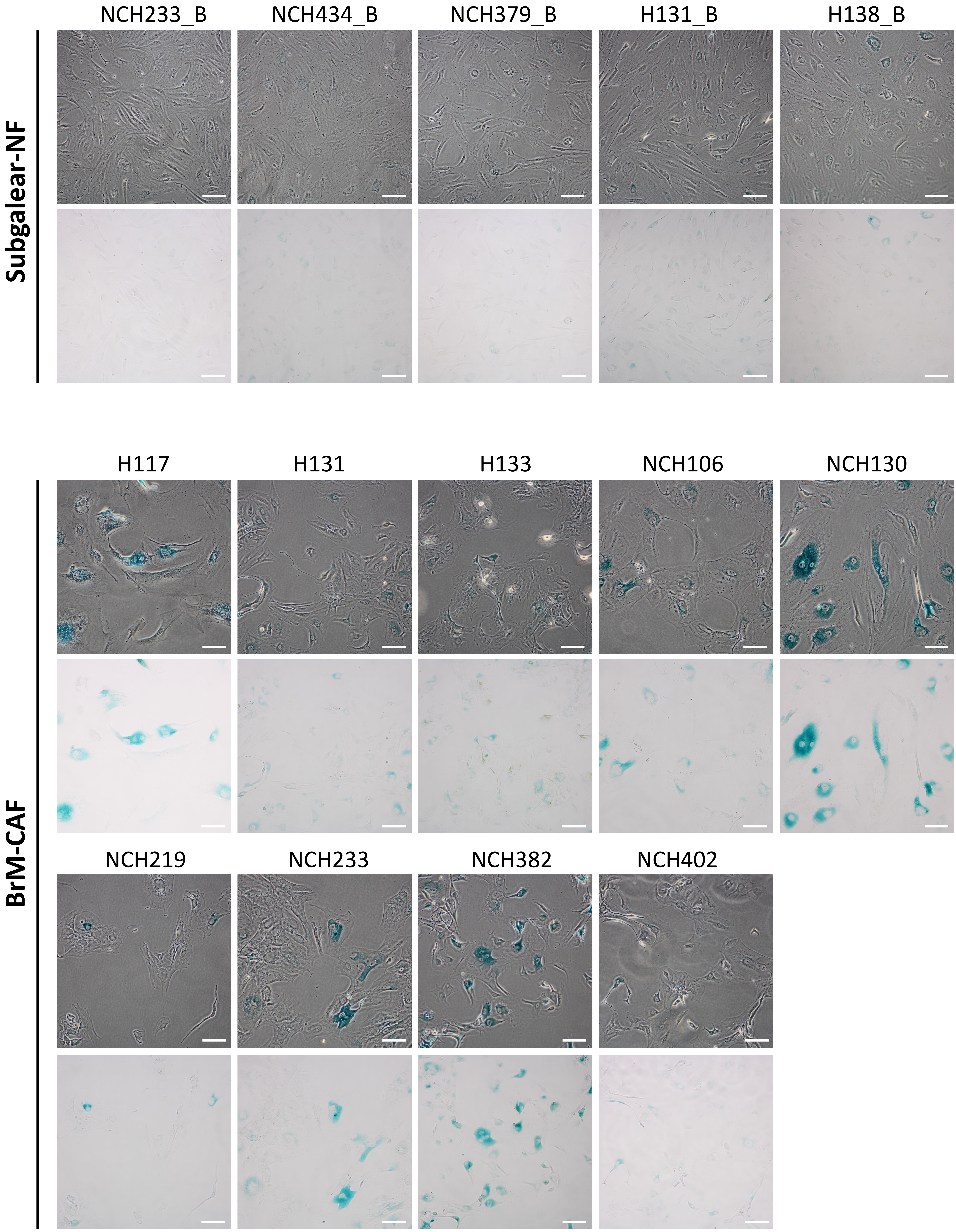

NCH233_B
NCH434_B
NCH379_B
H131_B
H138_B
Subgalear-NF
H117
H131
H133
NCH106
NCH130
NCH219
NCH233
NCH382
NCH402
BrM-CAF
