# Supplementary material for: Brain metastasis‐associated fibroblasts shape the tumour microenvironment to enhance cancer cell invasion
Source: Brain Pathol. 2026 Aug 4:e70129. Online ahead of print. doi: 10.1111/bpa.70129 (PMC13437050; doi:10.1111/bpa.70129)
Supplement: Supplementary file 8 [file BPA-9999-e70129-s014.pdf]

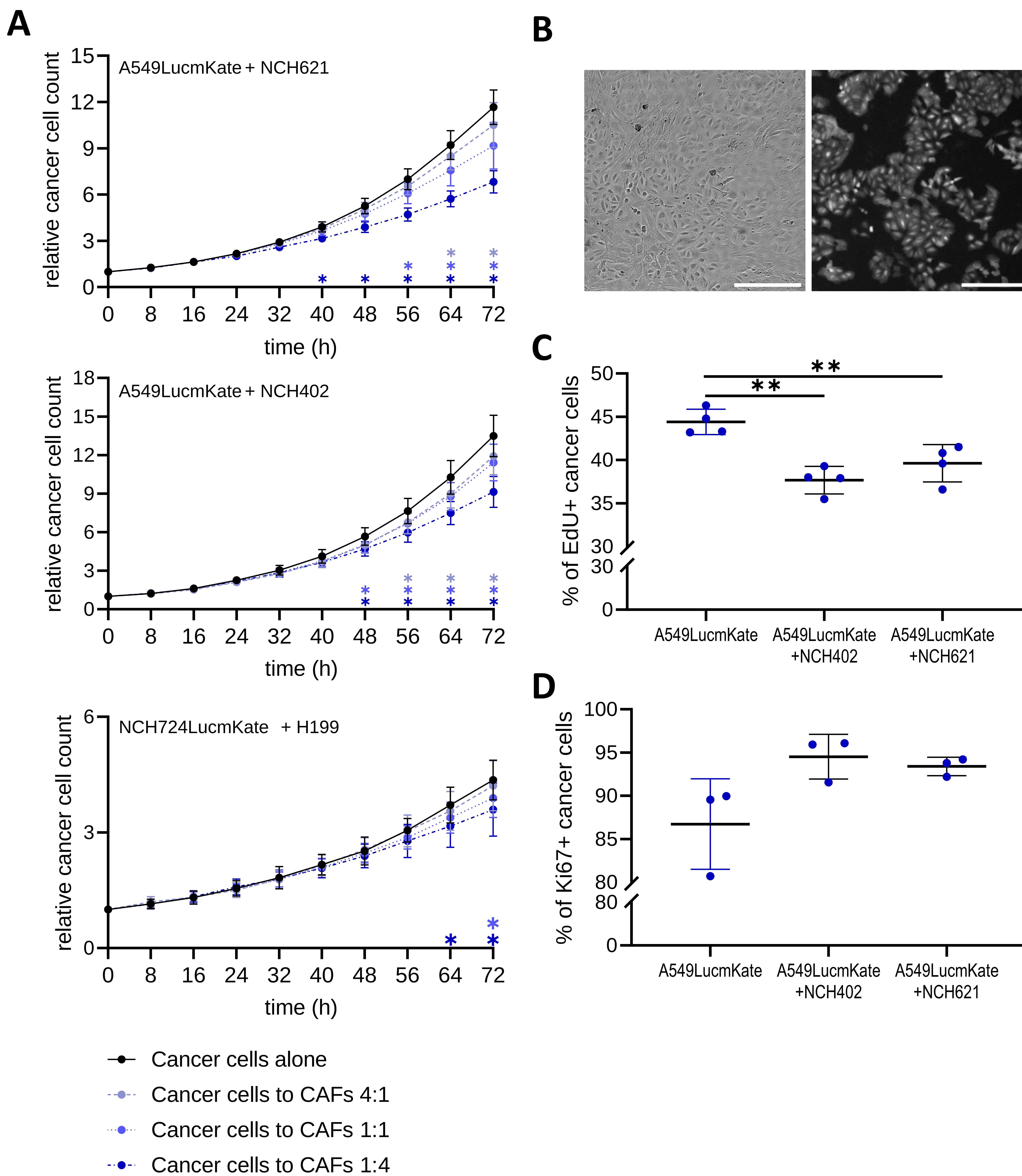
A
B
C
D
A549LucmKate + NCH621
A549LucmKate + NCH402
NCH724LucmKate + H199
relative cancer cell count
time (h)
% of EdU+ cancer cells
% of Ki67+ cancer cells
A549LucmKate
A549LucmKate +NCH402
A549LucmKate +NCH621
Cancer cells alone
Cancer cells to CAFs 4:1
Cancer cells to CAFs 1:1
Cancer cells to CAFs 1:4
