# Supplementary material for: Brain metastasis‐associated fibroblasts shape the tumour microenvironment to enhance cancer cell invasion
Source: Brain Pathol. 2026 Aug 4:e70129. Online ahead of print. doi: 10.1111/bpa.70129 (PMC13437050; doi:10.1111/bpa.70129)

BrM-patient derived cancer cell culture

Cell culture	Origin of the brain metastasis	FAP	TE7	αSMA	PDGFRβ	Vimentin	PCK	GFAP	CD45
NCH724	Lung	0%	0%	0%	0%	0%	100%	0%	0%

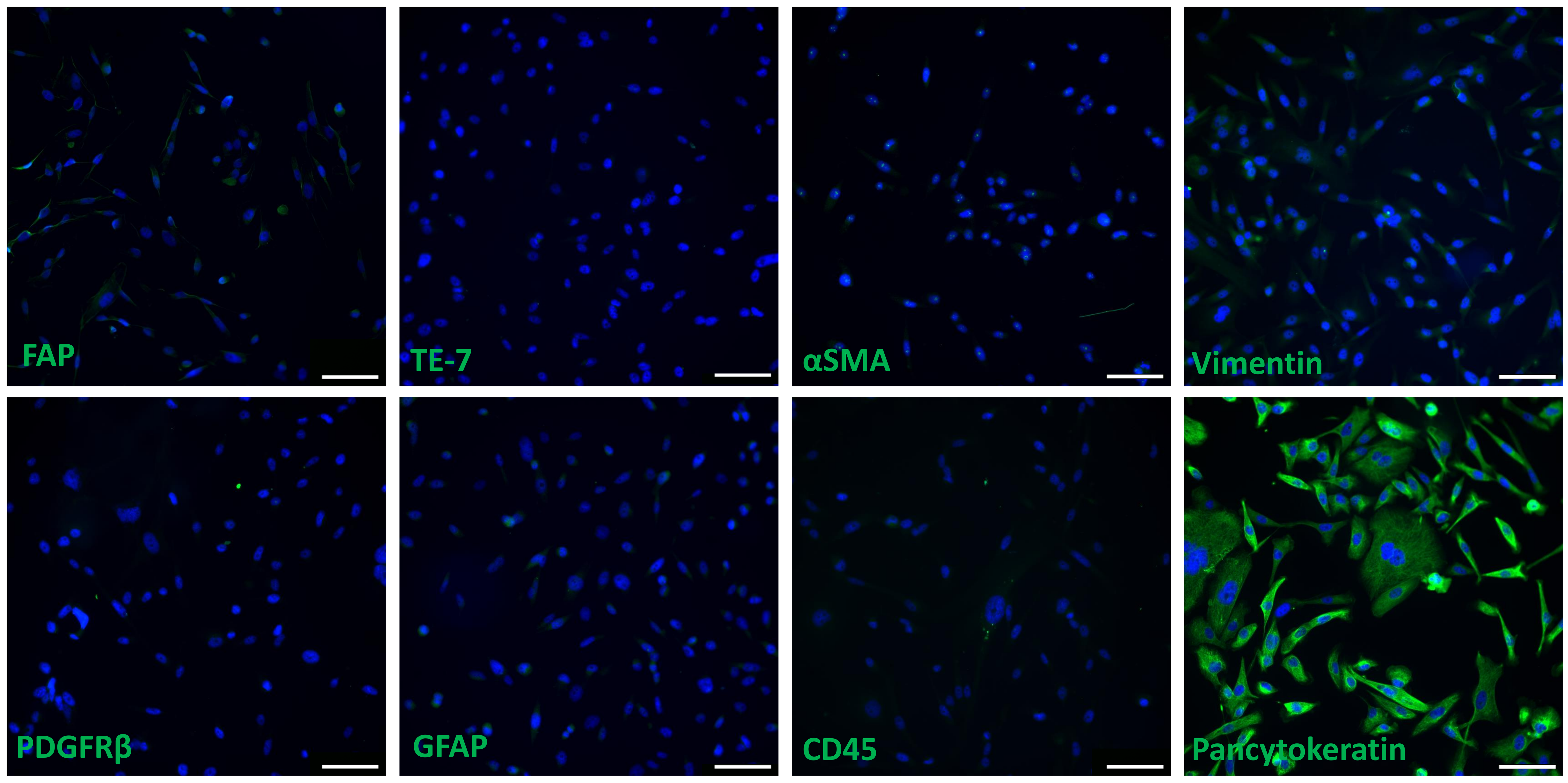

Supplement: Supplementary file 9 [file BPA-9999-e70129-s018.pdf]
